# Understanding the performance of county health service delivery in Kenya: a mixed-method analysis

**DOI:** 10.1093/heapol/czab129

**Published:** 2022-02-08

**Authors:** Wu Zeng, Anita Musiega, Joyce Oyasi, Laura Di Giorgio, Jane Chuma, Ruoyan Lu, Haksoon Ahn

**Affiliations:** 1 Department of International Health, School of Nursing & Health Studies, Georgetown University, 3700 Reservoir Rd NW, Washington, DC 20007, USA; 2 Health Economics Research Unit, KEMRI-Wellcome Trust Research Programme, 197 Lenana Place, Lenana Road, Nairobi, Kenya; 3 Centre for Economic and Social Research, Queensway House, Kaunda Street, Nairobi, Kenya; 4 World Bank, 1818 H Street NW, Washington, DC 20433, USA; 5 World Bank Kenya Office, Delta Center, Menengai Road, Nairobi, Kenya; 6 School of Public Health, Fujian Medical University, 1 Xuefu North Road, Newe University District, Fuzhou 350122, China; 7 School of Social Work, University of Maryland, 525 W. Redwood Street, Baltimore, MD 21201, USA

**Keywords:** Efficiency, health systems, Kenya

## Abstract

To better understand the wide variation of performance among county health systems in Kenya, this study investigated their performance determinants. We selected five counties with varied performance and examined their performance across five domains containing 10 thematic areas. We conducted a stakeholder analysis, consisting of focus group discussions and key informant interviews, and administered a quantitative survey to quantify the magnitude of inefficiency. The study found that a shortage of funding was one of the most common complaints from counties, leading to inefficiency in the health system. Another major reason for inefficiencies was the delay in disbursing funding to health facilities, which affected the procurement of medical supplies and commodities essential for delivering healthcare to the population. In addition, lack of autonomy in procuring commodities and equipment was repeatedly mentioned as a barrier to delivering quality health services. Other reported common concerns contributing to the performance of county health systems were the lack of lab tests and equipment, low willingness to join health insurance, rigid procurement policies and lengthy procurement process, lack of motivation and incentives for service delivery, and poor economic status. Despite the common concerns among the five counties, they differed in some schematic areas, such as the county’s commitment to health and community mobilization. In summary, this study suggests various factors that determine county health system performance. Given the multifaceted nature of inefficiency drivers, it is necessary to adopt a holistic approach to address the causes of inefficiencies and improve the county health systems

## Introduction

Kenya committed to providing Universal Health Coverage (UHC) to its citizens to improve their health status by 2022. To fulfil this goal, a series of health reforms have been carried out. The Kenyan National Hospital Insurance Fund (NHIF) established in 1966 has been reformed to expand insurance coverage. As of 2018, NHIF provides health insurance to 16.7% of the total population in Kenya (Barasa *et al*., 2018). Along with the expansion of the NHIF, the Kenyan national government abolished user fees at primary health care facilities and introduced free maternal healthcare services in public health facilities in 2013. In 2018, Kenya launched a UHC pilot in four counties, where households receive free healthcare services in all public health facilities. These initiatives are critical in Kenya’s progress towards UHC (Okech, 2017).

Despite the unprecedented achievements so far, the Kenyan government’s financial constraints create significant challenges in providing affordable quality healthcare services to its citizens. In the fiscal year 2018/2019, the health sector, including the national and county levels, shared only 5.4% of the national budget. The health sector budget ranked 6th among 10 sectors reported in a recent budget analysis (Institute of Economic Affairs, 2018), with the budget for health at about one-fifth of that for education. Expanding fiscal space for health is critical to ensure that Kenya moves towards UHC to achieve sustainable development goals by 2030. Both domestic and international experiences suggest that efficiency improvement is one of the viable approaches to narrow the financial gaps for UHC (Chuma *et al*., 2014; Dijkstra *et al*., 2017; Kioko, 2013). This suggestion is consistent with the World Health Organization’s advocacy to improve efficiency as a means to progress towards UHC (Chisholm and Evans, 2010).

To improve efficiency, Kenya has undergone a series of reforms over decades. One of the major reforms is the devolution of health service provision from the national level to the local level of government. In 2010, Kenyans overwhelmingly voted for a constitution that introduced a new governance framework with a national government and 47 county governments. Under the devolved system, the national government is responsible for health policy and national teaching and referral health facilities, while the county governments are entrusted with all functions related to health service delivery up to health facilities Level 5 (county referral hospitals). The devolved health system was expected to improve efficiency, stimulate innovation, improve access to and equity of services, and promote accountability and transparency in service delivery (Bossert and Beauvais, 2002). However, Barasa *et al*. (2017) found that the institutional design of the devolution process created a recentralization within decentralization, reassigning the authority and power that sub-county structures, such as hospitals, had pre-devolution to the county level, resulting in a significant reduction in hospital’s autonomy over some hospital functions (i.e. strategic management, finance, procurement, human resources and administration).

Empirical studies, using data envelopment analysis (DEA) and/or stochastic frontier analysis (SFA), show that there is a wide variation in the efficiency of county health systems after the devolution (Bundi, 2015; Kioko, 2013; Makheti, 2017). DEA and SFA are the two common approaches to evaluate the technical efficiency of decision-making units, using input and output measures and generating efficiency scores that ranged between 0 (the least efficient) and 1(the most efficient). More robust indicators measuring the efficiency, such as occupancy rate and budget execution rate, also suggested substantial inefficiencies in Kenya’s health system. For example, the hospital occupancy rate, on average, was estimated at slightly over 40% in 2015/2016 (Ministry of Health, World Health Organization, 2016), and development spending had an execution rate of 52.6% in 2015/2016 (Dijkstra *et al*., 2017).

The findings of these studies suggest that to sustain and implement its health strategies, Kenya needs to focus on enhancing health system efficiency. However, such studies do not provide concrete evidence on where inefficiencies are, nor do they offer direct and implementable recommendations to address inefficiencies in the health system. Furthermore, these studies used the same production frontier for each type of health facility, an assumption that may not be correct. Consequently, policymakers should be cautious of interpreting efficiency scores. Efficiency scores from DEA or SFA are not intuitively understandable for policymakers given the complexity of the production process of healthcare. There-fore, the efficiency scores are generally indicative (Zeng *et al*., 2016; 2012).

To better understand the inefficiencies at the county level and address the limitations from DEA or SFA-type efficiency studies, this study aims to complement a typical DEA or SFA study with an in-depth analysis of county health systems to explore causes of inefficiencies in five selected counties and identify potential mechanisms to improve their performance.

## Methods

### Analysis framework

**Figure 1:**
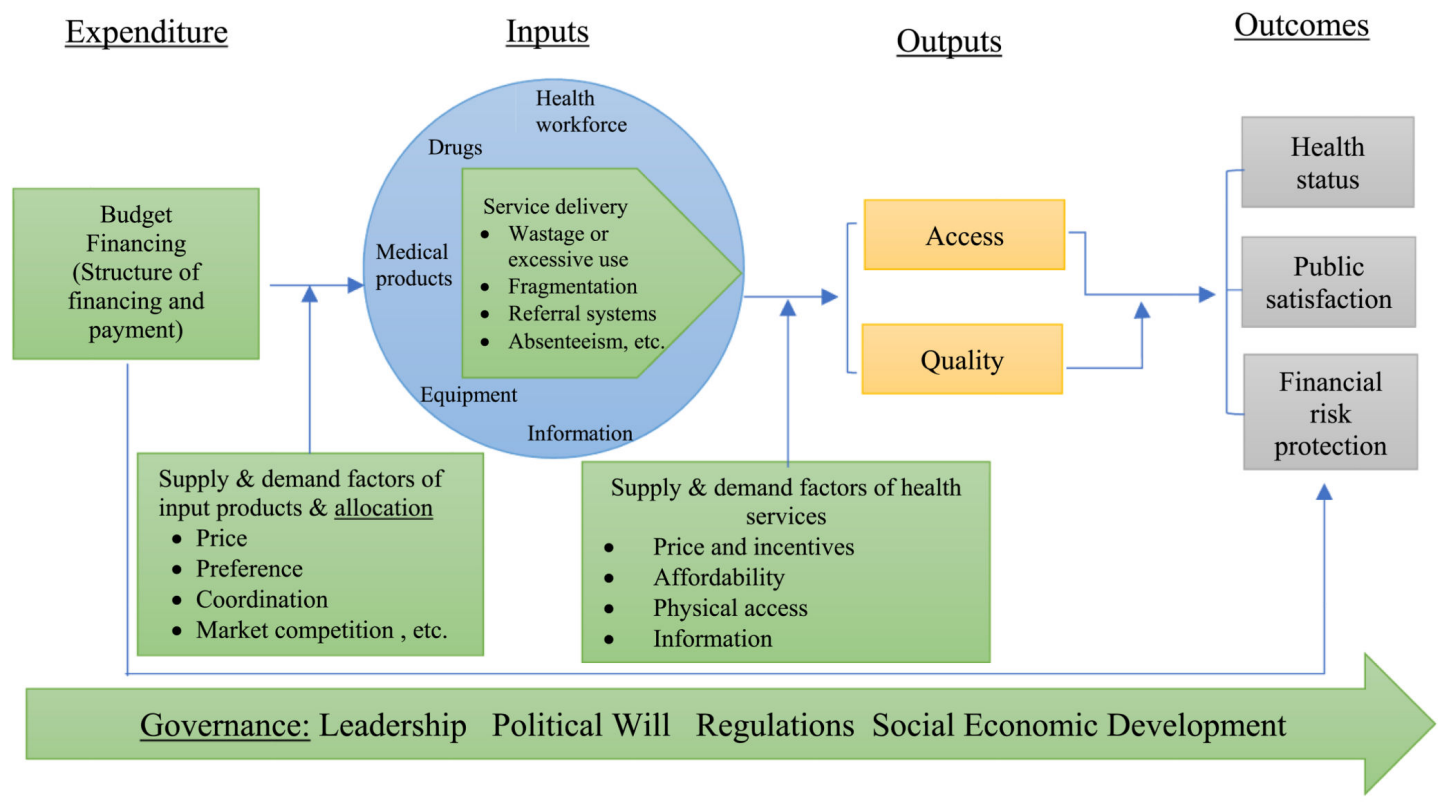
Theoretical framework to analyse causes of performance of health systems

To help understand the causes of inefficiencies in health systems, several frameworks have emerged in the last few years, including the health system framework (Cylus *et al*., 2016; World Health Organization, 2010) and input-output-outcome framework (Cylus *et al*., 2017). We used the framework developed by Zeng *et al*. (2020) for the analysis (see Figure 1). This framework extended the input-output-outcome framework (Jacobs *et al*., 2012) and combined the health system framework and input-output-output frame-work together, providing a more complete picture to explore drivers of health system performance. It included a total of five domains in which to examine the drivers of the performance of a health system: governance, budgeting and financing (payment mechanisms included), supply and demand factors of input productions and budget allocation, service delivery, and supply and demand factors of health service.

### Study design and sampling strategy

We purposively selected five counties for the study representing different levels of performance based on the efficiency scores from DEA and SFA (Korir and Chuma, 2018): Two counties (Counties 1 and 2) serve as high-performance counties, with an efficiency score of 94.21% and 90.58%, respectively; one medium-performance county (County 3), which had an efficiency score of 77.75%; and two low-performance counties (Counties 4 and 5), with an efficiency score of 49.21% and 43.93%, respectively. The names of the selected counties were blinded because the purpose of the study was to explore potential causes of counties’ performance rather than to rank their performance.

We used a qualitative approach to solicit opinions on drivers of county health system performance and a quantitative approach to measure the extent of the impact of determinants. This study comprised two major components: (1) a stakeholder analysis, consisting of focus group discussions (FGDs) and key informant interviews (KIIs), and (2) a quantitative stakeholder survey to assess the magnitude of inefficiency.

#### Stakeholder analysis

All counties had a similar health delivery system structure in Kenya, from Level 1 to Level 5, which were communities, dispensaries, health centres, sub-county hospitals and county referral hospitals, respectively. We conducted a stake-holder analysis consisting of FGDs and KII to county health management teams (CHMTs) and staff at service delivery points. We used FGDs and KIIs to gather information from key stakeholders of the county health system. The key stake-holders included the CHMT, which generally consisted of the county executive committee member for health, chief health officer, and county director for health and head of health-related departments (e.g. head of human resource and head of procurement), and staff of health facilities (e.g. hospitals, dispensaries and health centres). For each FGD, all available CHMT members were invited on the day of the visit, including director of health and heads of various health departments. Generally, there were 7-10 participants in an FGD. In counties where there were fewer than 10 CHMT members shown in the FGD, one FGD was conducted. If there were >10 members in the FGD in a county, then two FGDs were carried out. Additionally, we also selected CHMT members and health providers (e.g. hospitals, dispensaries and health centres) to attend KIIs based on emerging themes of (in)efficiencies. One hospital, two dispensaries and two health centres were randomly selected to be included in the study. The directors of health facilities were invited in the KIIs. For FGDs and KIIs, the interview guide was organized into five domains of inefficiencies, with additional interview questions on recommendations to address inefficiencies. The interview guide for FGDs and KIIs is provided in Supplementary Appendix 1. We worked with the county’s Department of Health (DoH) to identify appropriate participants for FGDs and KIIs. In general, participants for FGDs were county management health staff while participants of KIIs included directors of health hospitals, dispensaries and health centres in addition to some county management health staff. In total, we conducted eight FGDs and 58 KIIs.

#### Stakeholder survey

**Figure 2:**
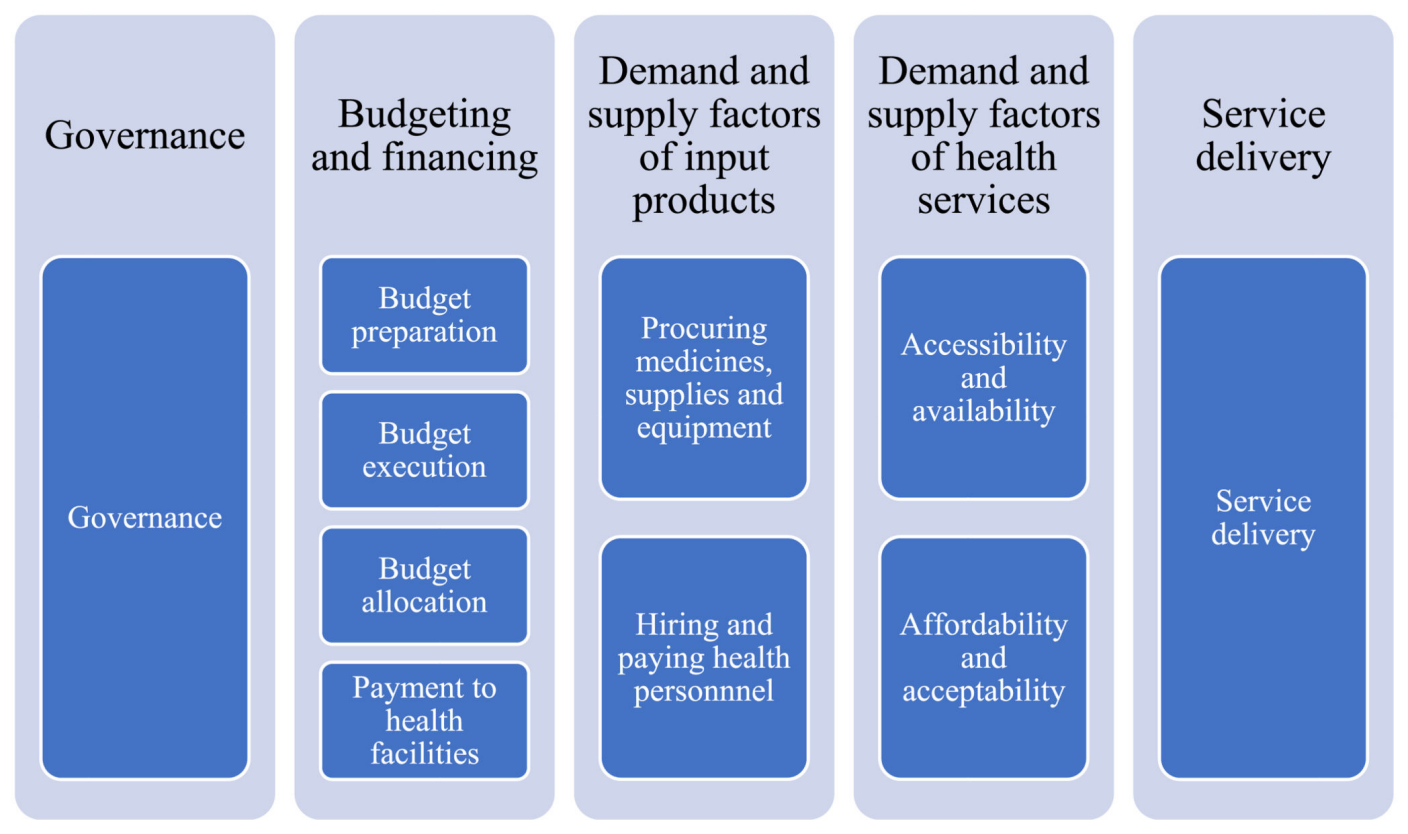
Five domains and associated 10 thematic areas

To measure the potential for performance improvement in the county health system, we developed survey instruments that were consistent with the qualitative interview guide. For participants to better identify the causes and magnitude of inefficiencies, we separated the five domains into 10 thematic areas. The relationship between the five domains and the 10 thematic areas is illustrated in Figure 2.

In the stakeholder survey, we first assessed the existence of inefficiencies in the 10 thematic areas using the Likert rating scale from 1 to 5 indicating ‘strongly disagree’ to ‘strongly agree’, with one Likert item per area. It is important to note that while Likert scales offer a straightforward way to quantify inefficiencies, they do not provide the most accurate method of estimation. A higher score indicated the presence of inefficiencies. We regarded a score above 3 (neutral) to indicate an inefficiency concern in that area. For areas identified as being affected by inefficiencies, we further prompted participants about in-depth causes and asked them to rank the magnitude of the inefficiencies ranging from 0 (no efficiency gains) to 5 (substantial efficiency gains) based on their perception. There were multiple items in each area. A simple average score was generated from the items in each area. A higher average score would suggest higher potential to achieve efficiency gains if the inefficiency were addressed and indicated a higher level of inefficiency. We used a cut-off of 3 to determine the magnitude of inefficiencies. A total of 104 individuals participated in the stakeholder surveys.

### Data collection

A local team collected quantitative and qualitative data from the five counties. The survey instruments and interview guide were piloted in Kajiado County in October 2018 and then were finalized based on the feedback from the pilot. Before the FDGs, KIIs and stakeholder survey were conducted, informed consent was obtained from each participant. FDGs and KIIs were recorded, and the stakeholder survey was collected.

### Data processing and analysis

A codebook was developed for data coding and cleaning. Quantitative data from the stakeholder survey were entered through Epi-info and converted into Microsoft Access. The data analysis was carried out using Stata version 15.0 (Stata Corporation, College Station, TX, USA). We calculated means and standard deviations for most variables and generated inefficiencies scores. Qualitative data from the audio recordings for the FGDs and KIIs were transcribed and imported into ATLAS.ti software (ATLAS.ti, Corvallis, OR, USA) to facilitate coding, categorizing and analysing. The initial codes were based on the interview guide. Priori codes were consistent with the five domains and 10 thematic areas. Under prior codes, sub-codes were developed to indicate whether the participants were neutral, positive or negative to the existence of potential inefficiency concerns. We used ATLAS.ti to apply the codes to the associated quotes. We then extracted and categorized quotes and analysed the potential relationship among them.

It should be noted that the stakeholder survey, FGDs and KIIs were conducted almost at the same time. Thus, findings from FGDs and KIIs might not necessarily cover all the inefficiency areas identified in the stakeholder survey.

## Results

### The profile of five counties

Table 1 provides the budget and human resource information and characteristics of the five counties. The average population size was 1.2 million per county: County 2 had the largest population of 2.2 million and County 3 the smallest population of 0.6 million. There were, on average, 217 health facilities per county. In all five counties, the administrative budget accounted for the largest share of the approved budget, ranging from 66.6% in County 2 to 84.6% in County 5. County 1 had the highest per capita budget with $34.30. On human resources, the number of staff, excluding community health workers (CHWs), was 1.2 per 1000 population on average.

Regarding output indicators (Table 2), the average inpatient admissions and outpatient visits were 18 672 and 899 490 per county, respectively. Consistent with population size, County 2 provided the highest number of health services combining inpatient admissions and outpatient visits. We further converted the inpatient admissions to outpatient visits equivalent, assuming the average length of stay per admission was 4 days (Department of Health, 2017), and one hospital day was equivalent to three outpatient visits (Shepard *et al*., 2000). The average outpatient visit equivalent per health staff was estimated to be 851 per staff.

### Inefficiencies from qualitative interviews and stakeholder survey

#### Overall inefficiency by thematic areas

Table 3 shows the inefficiency scores in the 10 thematic areas as reported through the stakeholder survey conducted in the five counties. Higher numbers correlate to relatively higher inefficiencies in the county. Overall, County 3 (the medium performer) had the highest average inefficiency score of 3.83 in the 10 areas and had the highest inefficiency scores for six areas (one area is tied with County 4). County 4, as the county with lower performance, had the second highest average inefficiency scores of 3.70, with the highest inefficiency in five areas (one area is tied with County 3). County 5 was next to County 4 with an average efficiency score of 3.26. County 1 and County 2 had the lowest average inefficiency scores of 3.09 and 2.13, respectively.

Below we provide a detailed analysis of inefficiency for each of the 10 thematic areas.

#### Inefficiencies in governance

Among the five counties, there was a wide variation of performance in the 13 items on governance (Supplementary Appendix Table A1). Implementation autonomy and political commitment were highlighted during the qualitative interview.

##### Lack of implementation autonomy

Since the devolution, counties enjoyed the autonomy that was given in budgeting and providing health services. However, interviewees felt that the constraint in autonomy was primarily at the health provider level regarding autonomy in procuring commodities and hiring professional staff. While health facilities sometimes were able to use their funds to purchase less expensive equipment or commodities, overall, they did not have the autonomy to procure pricy equipment and commodities. Health facilities were required to compile and submit their requirements to the county procurement department, which then procured and made payments on their behalf. The procurement process could be very long, sometimes due to procurement staff being unfamiliar with procurement products.

##### Commitment to health

Stakeholders had different views on the county’s commitment to health. In County 2, the commitment seemed high, which was reflected in the share of the county’s budget for health (36% of the total county budget). County 3, however, had contradictory opinions regarding its commitment to health. At the county-management level, stakeholders pointed out a previous disconnection between the County Assembly and the Health Executive as the County Assembly often opposed proposals from the DoH. The relationship between the two parties had improved since the new government came into power in 2017: funding for health had been increased, and the county government put health as the priority of the county’s development.

#### Inefficiencies in budget preparation, execution and allocation

From Supplementary Appendix Tables A2-A4, a lack of routinely collected cost data of inputs (i.e. costs of services delivery) for budgeting appeared to be prevalent in all counties except for County 4 on budget preparation; lengthy process to disburse funding, unavailability of funds and understaffing were significant concerns shown in at least four counties on budget execution; the political consideration for allocating funds for health was a concern prevalent in all five counties on budget allocation. In the qualitative interviews, a shortage of funding and human resource emerged as major inefficiency concerns.

##### Shortage of funding

Shortage of funding was one of the most common complaints from the counties, and one of the leading causes of inefficiency in the health system. Funding shortages often led to other inefficiency concerns, such as the shortage of human resources, lack of essential lab tests and equipment, and poor maintenance of equipment. All counties expressed the issue of funding shortages, although several counties stated that the budget had been significantly increased since the devolution. In County 2, the county budget for health was ∼36% of the total county budget. However, it is evident that a significant share of the health budget was devoted to paying salaries and allowances for health personnel. In County 3, ∼75% of the recurrent budget went to salaries and remuneration, leaving only 25% of the recurrent budget for service delivery.

One of the challenges, of course, is the budget. If we do not have enough budget, you cannot employ [staff], and start the process of determining how much budget is needed for health in terms of human resources. It seems that people do not have the capacity to come up with [such an estimate]. They do it on [a] historical basis (County 2).

##### Shortage of human resources

Another key barrier to executing the budget was the over-all shortage of staff. Although human resource management had improved substantially, stakeholders emphasized that the shortage of clinicians and nurses was the central issue in the county health system. This issue existed particularly in the lower level of the health system, such as dispensaries, where clinicians (i.e. nurses) experienced excessive workload. Besides the shortage of nurses, there was also a shortage of other specialists, such as physiotherapists, orthopaedic clinicians and medical engineers. The reasons for the shortage were multifaceted. The most common explanations included funding shortage and restricted hiring autonomy at health facilities. In addition, there were other reasons contributing to the shortage of clinical or supporting staff, such as the pursuit of further education, expansion of health facilities and lack of financial incentives. In County 2, medical personnel who had left posts to attend training or education had significantly affected the service delivery.

#### Inefficiency in paying health facilities

Payment mechanisms to health facilities are critical in improving health system efficiency. Supplementary Appendix Table A5 shows that paying health facilities was a concern for all five counties except County 4. Rigid payment mechanisms, delays in payment, the lack of financial incentives for providing better and more services affect at least four counties, while the latter two items were prominent during the qualitative interview.

##### Delays in payment

Delays in payment were repeatedly mentioned during the interviews in all five counties. They experienced delays in payment of salaries from the national treasury, and some experience delays in getting funding from conditional grants or the NHIF. Thus, there was a delay in paying for procurement of commodities, which resulted in an interruption in service provision, such as stock-outs of medicines.

Because we usually rely on the national treasury to release the funds to the Counties. So, you find that most of the time [national treasury] delays in dispersing the funds, so some-times we get a delay in procuring our health commodities. In such instances we get shortages but somehow, we try to curb by using the Facility Improvement Fund (FIF) for the bigger facilities (County 4).

The delay in payments seemed to result primarily from a shortage of funding in the national treasury.

##### Lack of motivation and incentives to provide services

Kenya uses three purchasing arrangements, including public integrated, public contract and private contract (Munge *et al*., 2017). In general, stakeholders found that the motivation to provide more and better health services was limited under the current payment mechanism. The shortage of staff overburdened the health system and exhausted the health personnel. However, there were no financial mechanisms to reward their hard work. The line-item budget did not incentivize health facilities to provide more services. Some stakeholders noted that the devolution had, in fact, demotivated health facilities for service provision, given that some health facilities were not allowed to collect user fees.

#### Inefficiencies in procuring medicines, supplies and equipment

The average inefficiency scores in the procurement of medicines, supplies and equipment are <3.0 (Supplementary Appendix Table A6) in all five counties. However, a few common issues arose in this area, including lack of procurement autonomy at health facilities and limited domestic production, which affects at least four counties.

##### Lack of procurement autonomy at health facilities

Autonomy in procurement was repeatedly mentioned as a barrier to implementing county strategies and work plans. Before devolution, procurement had been primarily conducted by the national agencies. Since devolution, the budgeting and procurement of high-value goods have been done by the county department of procurement. Still, the process does not necessarily filter down to the health facilities that request such goods. A notable complaint was that the user of goods (i.e. hospitals and health centres) was not involved in procurement throughout the process. Health facilities requested certain goods or products, but the county procurement unit might not know where to obtain them or did not understand the urgency of the need. Such centralization within decentralization has caused significant delays in providing requested goods to health facilities; they have even procured products not suitable for use.

##### Delays in the procurement of drugs and accumulated debts with suppliers

All five countries had complaints concerning delays in procuring and receiving drugs. The reasons for delays were multifaceted: (1) some delays were due primarily to a shortage of funding resulting in health facilities being in debt to their suppliers. Thus, suppliers refused to supply any drugs or shipped a reduced order; (2) delays could sometimes be caused by an irregular supply chain of the suppliers and the lack of communication between suppliers and health facilities; and (3) the shortage of pharmaceutical personnel and lack of capacity to quantify and forecast drug needs could lead to inaccurate projections in the health facilities and an over- or under-supply of drugs.

Sometimes there are delays and we are never quite sure when drugs will come. …You do an order; you don’t know when they’ll come. Maybe an order takes a month until the day you finish your payment as you had promised. … if we had no debts, then they supply very fast once you make your order. they do not communicate and tell us when the drugs will be in County 3 (County 3).

#### Inefficiency in hiring and paying staff

Supplementary Appendix Table A7 lists eight potential causes of inefficiency related to hiring and paying health personnel. Out of five counties, four had an average score >3.0, indicating potential concerns in hiring and paying health personnel. The average scores ranged from 2.33 in County 2 to 3.32 in County 3. Unclear staff-hiring policy, lack of autonomy in hiring/laying off staff, lack of transparency in the hiring process, lack of professional career development opportunities, and insufficient training and education opportunities were prevalent in at least four counties. The lack of autonomy in hiring/laying off staff was the key inefficiency concern in the qualitative interviews.

##### Lack of autonomy in hiring/laying off staff at health facilities

Health facilities did not have the authority to hire or lay off technical staff, although they have the autonomy to hire supporting staff, such as drivers and cleaners. The responsibility of hiring technical staff resided in the county public service board. The county DoH played a role in giving the public service board the parameters on hiring the number and type of staff, budget and cost implications. Sometimes, a disconnection among health facilities, the county DoH and the public service board led to (1) a mismatch of skills and (2) over- or under-hiring of staff. Additionally, PFM Act 2012 stipulates that the wage bill should not exceed 35% of domestic revenue (The National Treasury and Planning, 2018), aiming to leave more resources for service delivery. In counties that require investment in human resources due to health facility expansion, such a rigid hiring policy restricted the recruitment of needed human resources to deliver health services.

One [thing] we need to improve on human resources, the technical team in every department, we need to improve, like in this facility…we have a physiotherapist, we also need to have an occupational therapist, we also need to have a nutritionist, so that patients who come from those remote areas, with a simple issue like nutrition, we do not have to refer this patient to other facilities and it is an issue that can be handled in this facility (County 5).

#### Inefficiency in availability and accessibility of care

Supplementary Appendix Table A8 lists potential causes of inefficiency related to the availability and accessibility of the care. On availability and accessibility, four out of five counties had an average score <3.0. Three factors, insufficient resources (discussed previously), lack of essential lab tests and image services, and burdensome data reporting, were prevalent in at least four counties. During the stakeholder interviews, community mobilization emerged as a key inefficiency concern.

##### Community engagement

Most stakeholders realized the importance of community engagement in delivering quality health services to needed populations. However, the degree to which the community was engaged in health services seemed low. In County 5, despite developing a community health strategy, efforts to establish community units were insufficient according to the strategy and the work plan. There were only 44 community units at the time of the survey, compared to 80 community units that were supposed to be established. The high cost of establishing community units was the primary reason for the delay. Additionally, attrition of CHWs was more prevalent in County 5, jeopardizing the sustainability of the CHW programme. Additionally, CHWs were often volunteers without payment, and, as a result, their engagement in health delivery was irregular and not sustainable.

That is also a challenge, being an urban city… cosmopolitan city, we train the community health workers…because this is something that they are only volunteering, so when they get jobs they leave, but again issues of attrition are very high and we keep on selecting some new people, training them and when they get good jobs they leave. That is the challenge with urban places unlike when you go to rural because they are always there so the retention is very high (County 5).

#### Inefficiency in affordability and acceptability of care to the population

On affordability and acceptability, Counties 1 and 2 had an average score higher than 3.0 (Supplementary Appendix Table A9). The county with the lowest score was County 3, with an average score of 2.63. The lack of financial protection mechanisms and insufficient health education were prevalent in at least four counties. During the qualitative interview, the poor economic status of the population and low willingness to join health insurance were the major concerns.

##### The poor economic status of the population

In health facilities that charge user fees, such as sub-county hospitals or those ranked above that, affordability became an issue, particularly for the poor, although there was a waiver system. Some patients had to sell crops to seek care.

The greatest challenge for people not to afford health care are the charges. They are charged by the private facilities and the Coast General Hospital. Because they don’t have money, sometimes they just get paracetamols from her, not antibiotics (County 5).

In Kenya, Level 2 and Level 3 facilities are not allowed to charge user fees. Some patients were unable to distinguish the different levels of health facilities and did not expect to pay when seeking care in the higher level of facilities (i.e. sub-county hospitals). Even without user fees, transportation costs and productivity loss from seeking care could pose substantial financial barriers for the poor seeking healthcare.

##### Low willingness to join health insurance

Four counties scored higher than the cut-off of 3 in the survey question concerning the lack of financial protection mechanisms Kenya has a national health insurance scheme called the NHIF. It has a low enrolment rate: as of 2016, population coverage under NHIF was ∼16% of the total population (Barasa *et al*., 2018). Stakeholder survey ratings show NHIF has substantial room for improvement. People living in rural areas are not interested in joining the scheme, due to unfamiliarity with the benefits of NHIF, or adherence to their traditional beliefs.

That is where I still have a problem [on affordability] …these people, we have told them, you can register with NHIF, and pay 500 every month. It will be about kshs.6000 a year and you can get all the services plus your family. The willingness to register with NHIF is very low. Yes, it is very low (County 1).

#### Inefficiency in how health delivery system is organized

Supplementary Appendix Table A10 lists 18 indicators in this area. The average inefficiency score was rated from 2.23 in County 1 to 2.83 in County 2. Poor maintenance of equipment was a common cause of inefficiency that affected all five counties.

##### Lack of lab tests and equipment

Survey takers from several counties reported the lack of lab test kits and equipment, as well as problematic maintenance of equipment in health facilities. Sometimes, patients received care without needed lab tests and equipment examination, leading to misdiagnosis and low quality of care. Some health facilities have experienced difficulty in maintaining equipment and supplies of lab reagents. The shortage of equipment and lab tests was more salient at health centres/dispensaries. The challenge may rest on the lack of funds to purchase or delay in purchasing equipment/equipment parts and lab reagents, or the lack of capacity to perform the lab tests. Sometimes, the equipment was too old for maintenance.

In the lab we are missing several equipment. There is no centrifuge. The microscopes the person is using is not the standard because the clients now have to wait for a long time for him to process the specimen so that he can run the test to get the results (County 1).

## Discussions

This study examined potential causes of inefficiencies in five selected counties in Kenya and identified several common themes pertinent to the performance of those counties’ health systems The perceived common themes are as follows: (1) lack of implementation autonomy, (2) shortage of funding, (3) delays in payments, (4) rigid procurement policies and lengthy procurement process, (5) lack of autonomy in procurement and hiring, (6) shortage of personnel, (7) lack of motivation and incentives to provide services, (8) lack of lab tests and equipment and (9) poor county economic status. The main causes of these inefficiencies are poor economic development, lack of autonomy, funding limitations, and delays in payment and procurement.

One important factor that possibly differentiates county health performance is the county’s commitment to health. County 1 had the highest per capita health expenditure, and County 2 had the highest share of the county budget for health. These two counties were among the best performers in achieving health outcomes. Government financial commitment to health plays a vital role in ensuring needed services are provided at the appropriate levels. From the health financing perspective, the government contributed the most in financing health, accounting for 33.1% of the total health expenditure in 2015/2016 (Ministry of Health, 2019). Additionally, some private funding was also managed by the government, such as premiums collected through NHIF. From the health service delivery perspective, more than half of health services in Kenya are provided by public health facilities. As the country moves towards UHC, continued effort should be given to ensure that the government continues investing in healthcare.

Community engagement is perceived as another critical factor distinguishing county health performances. Studies conducted in Sri Lanka and Bangladesh show that the successful mobilization and use of CHWs are instrumental for an efficient health system (Chowdhury *et al*., 2013). Bangladesh uses much lower per capita health expenditure to achieve relatively good health outcomes. Its health system was regarded as one of the most efficient in the world (Chowdhury *et al*., 2013), and CHWs are the key to its success. In the five counties in this study, County 1 (the most efficient county) did well in mobilizing CHWs through establishing community units, but the other counties were not as successful. As all CHWs are volunteers, there has been a concern about the sustainability of the CHW programme. Developing a payment mechanism for them and monitoring their performance to hold them accountable for their work would help formally include them in the health system and improve their retention.

Poor economic status is regarded as one of the root causes for the inefficiency of county health systems Kenya’s GDP per capita was $1507 in 2017 (World Bank, 2019). Despite an impressive achievement in economic growth and a 10.5% drop in the poverty rate in the last decade, 36.1% of Kenya’s population was living in poverty in 2018 (Kenya National Bureau of Statistics, 2019). This study shows that Kenya’s health system inefficiencies were mainly caused by financial issues, including a shortage of funding, payment delays and shortage of input factors (e.g. staff, equipment and commodities). It is critical that the financial issues affecting the health system are addressed.

Efforts to improve economic development would address not only supply-related concerns but also concerns related to demand for healthcare services. For example, it is necessary to improve patient’s affordability and health education, as well as more general issues like road conditions and remote access to facilities. With the government’s commitment to ensuring that Kenya attains UHC, a fast-growing economy would accelerate progress towards this goal.

Respondents reported that many health facilities experienced delays in receiving funding from the Treasury. In County 4, health staff experienced significant delays in receiving salary payments. This finding is consistent with a public expenditure review conducted in 2014 (World Bank, 2014). The delay in payment primarily occurred in the national government. The key reason could be underfunding from the National Treasury. Additionally, this study shows that some delays are due to the misalignment of the budget. A timely and proper budgeting process should be employed to ensure resources are budgeted according to needs. Employing a budgeting process in the lower level of health facilities would help generate timely and adequate allocation of funding. Delays due to financial mismanagement may be corrected by streamlining the financial management system, at both the national and county levels.

Despite the devolution reform in Kenya, lack of autonomy in procurement and hiring seems to continue impeding efficient health service delivery. A study in a Kenyan hospital showed that devolution at the county level reduced the autonomy of the hospital, compromised the quality of services and reduced motivation among hospital staff (Barasa *et al*., 2017). Kenya transitioned into a devolved system of government in 2013, bringing greater autonomy for county governments. They are responsible for budgeting, procuring commodities and equipment, hiring personnel, and providing health services. One of the purposes of devolution was to hold the counties accountable for their health activities to improve the health sector’s performance. However, health facilities have little autonomy in procuring large-value commodities or equipment, as well as hiring personnel. The county department of procurement is responsible for procurement, while the county public service board is in charge of hiring. The disconnection between the users and procurers resulted in inaccuracies and omissions in procuring drugs and other supplies, given their different priorities. It is critical to streamline the procurement and hiring processes by engaging commodities and health facilities users that request personnel. As users of commodities are familiar with the needed items and are aware of the urgency for service delivery, this change in strategy could expedite the procurement process.

The interviewees in the study expressed a widespread concern of the shortage of health staff in health facilities. Although the current critical shortage is for nurses, some counties are also in great need of specialists, supporting personnel and CHWs. The shortage of health human resources is consistent with Wakaba’s study on the nursing workforce in Kenya (Wakaba *et al*., 2014). However, the reasons for the shortage vary substantially, such as the shortage of funding, rigid regulation of using funding for recruitment, lack of autonomy in hiring and laying off personnel, lack of financial incentives, expansion of health facilities, harsh working environment (i.e. remoteness and overburden with work), absenteeism from the post for education and so on. While it is exceedingly difficult to design an intervention that solves all these problems, some of the interventions that we propose to address the shortage of funding and autonomy of hiring could, to some degree, reduce the shortage of health personnel. More interventions tailored to the causes of the shortages should be designed. For example, in County 1, the human resource shortage seems to be primarily driven by the expansion of health facilities and a budget that could not keep up with the need to staff the facilities. Removing hiring regulation barriers (wage bill limit) and advocating for a larger health personnel budget should be the priorities. In the circumstance of severe shortage, some counties use contracted health personnel.

An additional concern was that both health facilities and health staff were not adequately motivated in providing health services. This concern is probably due to the lack of incentives (monetary or non-monetary). At the health-facility level, line-item budgets place significant limits on the autonomy of health providers to respond to urgent needs of the population, particularly for dispensaries and health centres where the government’s line-time budget is the major source of funding. Switching from a line-item budget to a programme-based budget or global budget, in combination with other mechanisms (e.g. with or without budget cap), should be considered to improve the autonomy of health providers in the financial management. To encourage health facilities to provide essential maternal and health services, a performance-based financing programme was implemented in some counties in Kenya (RBFHealth, 2014). The programme also faced challenges, such as lengthy verification of service delivery and delays in payment. Further investigation to overcome the barriers and reinforce a positive impact of the results-based payment is needed. Obadha *et al*. (2019) have provided more detailed recommendations on provider payment mechanisms in Kenya.

In summary, as Kenya is moving towards UHC, it is critical that county health systems maximize the benefits of the devolution reform and address bottlenecks of health system efficiency concerns that impede the counties from achieving their health goals. This study shed light on areas that could be further strengthened.

A few limitations of this study should be acknowledged. Firstly, the stakeholder survey and FGDs and KIIs were conducted almost simultaneously and thus we could not use the stakeholder survey results to inform FGDs and KIIs. Thus, findings from FGDs and KIIs might not necessarily cover all the inefficiency areas identified in the stakeholder survey. Secondly, some inefficiency items are not mutually exclusive to a certain domain, and there exist some duplications when examining inefficiencies. Thirdly, due to the complexity of the inefficiencies of health systems, we were not able to completely link county’s performance to its potential drivers. In this study, we singled out two factors (commitment to health and community engagement) that may contribute to the county’ health performance. There might exist other factors that affect their performance. Due to the exploratory and primarily qualitative nature of the study, this study may miss some other factors that differentiate the health performance among counties.

## Supplementary Material

Supplementary data are available at *Health Policy and Planning* online.

Appendix

## Figures and Tables

**Table 1:** Breakdown of budget and human resource and characteristics of five counties in USD

				Budget (per capita in USD)	Budget (relative amount)				Share of human resources		
County	Efficiency (%)	County population	Number of health facilities	Total	Administrative (%)	Hospitals (%)	Health centres/dispensaries (%)	Health personnel per 1000 population	Hospitals (%)	Health centres (%)	Dispensaries (%)
1	94.21	876 527	117	34.34	77.8	21.0	1.2	1.26	57.2	15.3	27.4
2^a^	90.58	2 250 367	497	27.49	66.6	29.9	3.5	0.91	60.3	39.7	–
3^b^	77.75	632 154	110	31.96	79.7	17.2	3.1	1.65	64.3	19.0	16.6
4	49.21	1 194 284	160	18.04	75.8	21.5	2.8	0.80	64.2	17.5	18.4
5	43.93	1 214 997	201	23.86	84.6	9.6	5.8	1.38	74.3	7.3	18.3
Mean	71.14	1 233 666	217	23.10	76.9	19.8	3.3	1.20	64.1	19.8	20.2

a Human resources for primary care (health centres and dispensaries) in County 2 could not be further broken down; thus, the number of personnel for health centres represents the sum of that for both health centres and dispensaries.

b The total budget was obtained from county financial officers. However, the budget breakdown was estimated based on the detailed budget items. For items that could be used for both hospitals and primary care, the average share from the remaining four counties was used. The breakdown of the development budget was proportional to the recurrent budget. The administrative budget includes salaries for all personnel in the county and budget for travel, utilities, stationaries, training, equipment and maintenance at the county-administrative level.

**Table 2:** Outputs of health facilities

	Inpatient admissions				Outpatient visits				Full immunization of children (FIC)				Skilled birth attendance					
County	Hospital	Health centre	Dispensary	Total	Hospital	Health centre	Dispensary	Total	Hospital	Health centre	Dispensary	Coverage	Hospital	Health centre	Dispensary	Coverage	Outpatient visits equivalent^a^	Outpatient visit equivalent/staff
1	8434	3059	283	11 776	135 445	237 977	761 239	1 134 661	2536	2536	13 531	85%	5652	6227	6487	55%	1 275 973	1159
2	26 813	3230	677	30 720	522 622	412 581	633 495	1 568 698	6751	8400	15 099	83%	11 367	5441	1383	70%	1 937 338	951
3	16 800	1348	15	18 163	279 210	335 264	229 657	844 131	2446	3586	2245	77%	5412	1468	38	79%	1 062 087	1021
4	13 168	2678	343	16 189	104 011	197 234	257 556	558 801	3384	8540	10 426	51%	5553	4075	1447	40%	753 069	787
5	14 623	1774	117	16 514	88 000	153 240	149 918	391 158	3035	6120	8536	81%	11 867	6204	767	78%	589 326	352
Mean	15 968	2418	287	18 672	225 858	267 259	406 373	899 490	3630	5836	9967	75%	7970	4683	2024	64%	1 123 559	825

a Assumption: assuming the average length of stay is 4 days/admission, and one hospital day is equivalent to three outpatient visits. SBA denotes skilled birth attendant; FIC denotes full immunization coverage.

**Table 3:** Existence of inefficiency in the 10 domains in five counties

Thematic areas	County 1	County 2	County 3	County 4	County 5
Governance	**2.94**	3.00	3.96	4.15	3.11
Budget development	3.28	**2.88**	3.89	3.55	**2.89**
Budget execution	3.47	3.13	4.15	4.15	3.71
Budget allocation	3.75	3.25	4.14	3.53	3.45
Paying health facilities	**2.89**	3.00	3.88	4.20	3.38
Procuring medicine, supplies and equipment	3.00	3.00	3.78	4.00	3.14
Hiring and paying health personnel	3.29	3.38	4.07	3.43	3.72
Making health services available and accessible	**2.72**	3.25	3.44	3.45	3.00
Providing affordable and acceptable services	**2.65**	3.25	3.46	3.38	3.21
Organization of health delivery system	**2.94**	3.13	3.56	3.21	3.00
Mean score	3.09	3.13	3.83	3.70	3.26

Note: The numbers in bold suggest no inefficiency concern, and numbers in regular font suggest the existence of potential inefficiency concerns.

## Data Availability

The data underlying this article were owned by the World Bank. Data will be shared upon request to the corresponding author with permission of the World Bank.

## Key messages

- Inefficiency remains a concern in county health systems in Kenya.
- The causes of inefficiency are multifaceted. Public financial management, including a shortage of funding, lack of autonomy in procurement and delays in payment, is one of the main drivers of the inefficiency in Kenya’s county health systems.
- Given the multifaceted nature of inefficiency drivers, it is necessary to adopt a holistic approach to address inefficiency concerns to improve the county health systems.
