# Supplementary material for: Understanding the performance of county health service delivery in Kenya: a mixed-method analysis
Source: Health Policy Plan. Author manuscript; Available in PMC 2022 Aug 26. (PMC7613432; doi:10.1093/heapol/czab129)
Supplement: Appendix [file EMS152546-supplement-Appendix.docx]

# Appendix 1. Instruments for FGDs, KIIs and stakeholder survey

**Informed Consent Form**

**Study Title**: Improving performance of county health service delivery in Kenya: A critical analysis of the factors affecting their performance and potential mechanisms for improvement

**Part I: Information Sheet**

Since the start of Kenya’s health system devolution to the county level in 2013, the county’s Department of Health has taken the major responsibility of budgeting, planning, and providing services. A recent study shows that counties are performing differently from each other in regard to service delivery However, there is little information on why some counties are performing better than others.

Funded by the World Bank, Brandeis University is conducting this study, which aims to document the factors that contribute to or hinder performance of county health systems: what works well, what does not, and what can be done to improve the performance of the health sector at the county level. This information will contribute towards better understanding of what drives performance in Kenya’s health sector at the county level, and will inform policy recommendations for performance improvement.

You are being invited to take part in this research project because your experiences with county health systems and your familiarity of health service delivery systems in Kenya would provide valuable insights for us to understand the performance of counties’ health system in Kenya.

During this study, you will be asked to participate in focus group discussions (FGDs) or key informant interviews (KIIs), and a stakeholder survey. Each FGD and KII will last about 90 minutes. The researcher will ask you a series of questions to get your insights on causes of performance in your county health system. For FGDs, you will be placed in a group of about 8 participants, while you will be an individual interviewee for KIIs. During the interview and discussion, we will record the conversation to allow us accurately to capture and transcribe your opinions. For the stakeholder survey, you will be asked to complete a survey assessing the magnitude and causes of inefficiencies in the county health system. The survey is expected to take 40 minutes.

We do not foresee any risks to you as we are more interested in understanding your opinions on health services delivery in your county. You do not have to answer any question or take part in the survey if you feel the question(s) makes you uncomfortable.

We will not be sharing information about you to anyone outside of the research team. The information that we collect from this research project will be kept private. All the personal information such as your name will be kept safely. All entered data will be password protected.

**Part II: Certificate of Consent**

I have read the above information or it has been read to me. I have had the opportunity to ask questions and any questions I have been asked and have been answered to my satisfaction. I consent voluntarily to be a participant in this study.

Print Name of Participant: ________________________ Signature of Participant: __________________

DD/MM/YYYY:______________________________________

Interview guide for focus group discussion (FGDs)

Objectives: This interview guide aims at obtaining insights on factors affecting performance of Kenya’s county health systems. The questions below will help us deepen the understanding of health systems’ barriers, in order to enhance the service delivery at the county level.

Participants: FDG participants are expected to be county financiers and regulators primarily at the county level, such as county government health officials.

Below we provide a generic interview guide for this study for researchers’ use. However, depending on the expertise and responses of interviewees, researchers should choose the most relevant questions to obtain their expert opinions.

Note: When asking questions, please organize questions in an order as such: Description of situation, stakeholder’s view of performance (major challenges and opportunities), exploration of causes of performance, suggestions to improve the performance, and feasibility of the suggestions for the improvement.

**Part I. Generic information of participants (Researcher should note down the information from each participant in the FDGs or KIIs)**

County name: ___________________________Department or organization ___________________________

Position: ____________________________ Years in position (yrs): ____________

**Part II. For focus group discussions (FDGs)**

**General options on performance of health system performance and potential causes.**

**Guide:** Provide a brief introduction of the purpose of this study, and then ask participants to provide their assessment of the county health systems’ performance, the key challenges of performance, causes of challenges, potential interventions to address the challenges and causes, and feasibility of the proposed interventions. Some example questions are:

1. Could you provide a brief overview of health systems in your county (Number and size of governance bodies, hospitals, clinics, referral systems, health programs, human resources, etc.)?
2. In your opinion, is your county health system operating with full efficiency/highest performance to improve population’s health?
3. On a scale of 0 to 10, how would you rate the performance of the health systems in your county? 0 means the health system does not function at all, while 10 means it functions very well and no further improvement could be made. What are some of the factors you considered in rating your county at that level?
4. In your opinion, what has been done well at the county management level (e.g. budgeting, procurement, regulation and coordination, governance and leadership, policy implementation. transparency and accountability etc.), and how does it affect the performance? (if interviewees mention specific areas, please explore further using questions for specific areas for KIIs as a guide)
5. In your opinion, what are the key challenges regarding the performance of the health systems at the county management level, and how do these challenges affect performance? Please provide specific examples. Explore root cause/reasons of challenges. (if interviewees mention specific areas, please explore further using questions for specific areas for KIIs as a guide)
6. In your opinion, what has been done well at the service delivery level (e.g. skill mix of personnel, motivation of personnel, availability of drugs and equipment, utilization of information, supply chain, community mobilizations, etc.), and how does it affect the performance? (if interviewees mention specific areas, please explore further using questions for specific areas for KIIs as a guide)
7. In your opinion, what are major challenges regarding the performance of the health systems at the service delivery level, and how do these challenges affect performance? Please provide specific examples. Explore root cause/reasons of challenges. (if interviewees mention specific areas, please explore further using questions for specific areas for KIIs as a guide)
8. What are your suggestions to improve the efficiency of your county health system?
9. How feasible is your suggestion to make changes and why?

Interview guide for key informant interviews (KIIs)

Objectives: This interview guide aims at obtaining detailed insights on factors driving performance differences in Kenya’s county health systems. The questions below will help us deepen the understanding of health systems’ barriers in order to enhance service delivery at the county level.

Below we provide a generic interview guide for this study for researchers’ use. However, depending on the expertise and responses of interviewees, researchers should choose the most relevant questions to explore to obtain their expert opinions.

Note: When asking questions, please organize questions in an order as such: Description of situation, stakeholder’s view of performance (major challenges and opportunities), exploration of causes of performance, suggestions to improve the performance, and feasibility of the suggestions for the improvement.

**I. KIIs for Director of Health or county health management team (CHMT)**

1. **Questions on health systems governance and leadership**

Governance in the health sector refers to a wide range of steering and rule-making related functions carried out by governments/decisions makers as they seek to achieve national health policy objectives that are conducive to universal health coverage.

1. Could you describe to me the governance structure for health in your county?
   1. What is the composition of the health leadership group and CHMT?
   2. How many people constitute the CHMT?
   3. What are the roles and responsibilities of the health leadership group, CHMT and related departments?
2. Governance generally concerns health-related policy implementation, regulation and coordination, and transparency and accountability. In your opinion, is the current governance structure for health functioning well? If yes, why do you say so? If not, why?
3. On a scale of 0 to 10, how would you rate the health governance function in your county? 0 means the health governance does not function at all, while 10 means it functions very well and no further improvement could be made.
4. In your opinion, what has been done well regarding the governance for health in your county and why? **Please see detailed questions A1, A2, A3, and A4 for specifics when interviewees mention specific governance areas**
5. In your opinion, what are the major challenges on governance in health in your county, and please provide specific examples? **Please see detailed questions A1, A2, A3, and A4 for specifics when interviewees mention specific governance areas**
6. What are your suggestions to improve the health governance in your county?
7. How feasible is your suggestion to make changes and why?

**A.1. Questions on priority setting, health strategy development, and strategy implementation**

1. What are the county’s health priorities? How are these priorities set?
2. In your opinion, are these priorities set according to health needs in your county? If yes, why? If not, what should be prioritized and why?
3. Has the county government developed specific health strategies and associated workplans on health that are geared to implementing National Government policies? Please explore
   1. Are strategies and workplans developed timely? (e.g. health financing, human resources; drug, medical supplies and equipment procurement; information systems; health delivery; private sector, clinical guidelines, and research)
   2. Are any critical policies missing? If yes, why do you think they are critical?
   3. Are health financing strategies, human resource strategies, maternal and child health strategies implemented on schedule?
   4. Are health departments able to effectively engage stakeholders, mobilize and deploy resources, and reconcile differences?
4. On a scale of 0 to 10, how would you rate the priority setting and health strategy development in your county?
5. In your opinion, what has been done well regarding priority setting and policy implementation?
   1. How does it affect the performance?
6. In your opinion, what are major challenges regarding priority setting and health strategy development?
   1. How do those challenges affect the performance?
7. What is your suggestion to improve priority setting and health strategy development?
8. How feasible is your suggestion to make changes and why?

**A.2. Question related to transparency and accountability**

1. Currently, what has CHMT been doing on improving transparency and accountability? Please explore:
   1. Does each member of the CHMT and health-related departments have clear division of roles and responsibility? If yes, is such division functioning well? If not, what are unclarities and how these unclarities affect the performance?
   2. Do the CHMT and relevant departments provide regular supportive/monitory supervision to health facilities to improve their performance? If yes, how often and how well have they performed? If not, why is the case?
2. On a scale of 0 to 10, how would you rate the transparency and accountability in your county?
3. What has been done well in improving the transparency and accountability?
   1. How does it affect the performance?
4. What are major challenges in improving transparency and accountability?
   1. How do these challenges affect the performance?
5. What is your suggestion to improve transparency and accountability?
6. How feasible is your suggestion to make changes and why?

**A.3. Question related to regulation and coordination**

1. In your county, what mechanisms do you have to regulate different organizations within the health systems? Please pay attention to decentralization, and explore regulations for the following:
   1. Public hospitals and clinicians (e.g. licensing, and hiring clinicians)
   2. Private hospitals and clinicians (e.g. licensing and hiring clinicians, contracting with government)
   3. Vertical programs (e.g. TB, HIV, malaria, program selection, financial and reporting regulations)
   4. Pharmacies and pharmaceutical companies (e.g. licensing, quality assurance, and price setting)
   5. Quality assurance agencies, how does it work?
2. In your county, how does the coordination take place among health-related departments and with other sectors (such as the private sector)? Explore.
   1. Does the county have a coordination institution to coordinate work within the health sector, and how does it work?
   2. Does the CHMT regularly communicate with Sub-CHMT and health facilities, and provide supportive supervision? If it does, how does it work?
   3. Does the county have a health stakeholder forum? If it does, how does it work? If it does not, how does the county coordinate the work with other organizations, such as donor communities?
   4. How effective is the coordination? Please ask for specific examples.
3. On a scale of 0 to 10, how would you rate the performance of regulation and coordination, respectively, in your county?
4. What has been done well in regulating health-related organizations/institutions and coordinating within or outside the health sector?
   1. How does it affect the performance?
5. What are major challenges in regulating health-related organizations/institutions and coordinating within or outside the health sector?
   1. How do these challenges affect the performance?
6. What is your suggestion to improve regulation and coordination?
7. How feasible is your suggestion to make changes and why?

**A4. Questions related to leadership**

1. In your county, how is the leadership for health, including CHMT, selected? Please explore:
   1. How many people are in the CHMT, and what is CHMT’s role in health management in your county?
   2. How are members of CHMT selected?
   3. How does CHMT manage the daily activities in your county?
   4. How effective is the CHMT, in your opinion, in managing health issues in your county (e.g. ability to inspire and motivate others, build strong teams, and solve complex issues)
2. On a scale of 0 to 10, how would you rate the leadership in your county?
3. What has been done well in improving leadership in your county?
   1. How does it affect the performance?
4. What are major challenges regarding the leadership in your county?
   1. How do these challenges affect the performance?
5. What is your suggestion to improve the leadership in your county?
6. How feasible is your suggestion to make changes and why?

**II. KII for procurement department manager**

1. **Procurement (health personnel, drugs, medical supplies, and equipment) and distribution (supply chain):**

The procurement here focuses on drugs, medical supplies, equipment, and human resources (e.g. medical doctors and nurses)

**B1. Questions related to procurement of drugs, medical supplies, and equipment**

1. Could you describe the procurement process for drugs, medical supplies, and equipment in your county? Please explore:
   1. Existence, size and composition of the procurement committee
   2. Procurement process (who, what, how, why, where, and when about procurement, including the determination of types of products and the amount of drug, medical supplies and equipment, negotiation process, and payment)
      1. What is the main source of drugs, medical supplies and equipment? Where does the county procure drugs, medical supplies and equipment from? Is KEMSA your main supplier? Besides KEMSA, are there other suppliers of drugs in your counties?
      2. How are suppliers selected? How do you ensure quality of the commodities? How are procurement prices determined for drugs, medical supplies, and equipment?
   3. How effective is the existing procurement process (e.g. over procurement, under procurement, mis-procurement, procurement at a high price, and procurement of inferior products)?
2. On a scale from 0 to 10, how would you rate the procurement process in your county?
3. What has been done well regarding the procurement of medicine, medical supplies and equipment in your county? (i.e. devolution and autonomy)
   1. How does it affect the performance?
4. What are major challenges regarding the procurement of drugs, medical supplies, and equipment?
   1. How do these challenges affect the performance?
5. What is your suggestion to improve the procurement in your county?
6. How feasible is your suggestion to make changes and why?

**B2. Questions related to supply chain**

1. Please describe how drugs, medical supplies, and equipment are distributed (supply chain) within the county. Please explore:
   1. Is there a central distribution center in your county to distribute drugs? If yes, how often does it distribute the drugs?
   2. How does the requisition of drugs collected and aggregated at the county level?
   3. How does the county determine the frequency and amount of medicine for distribution? Has the county used electronic information systems to make informed decisions to distribute medical products and drugs?
   4. Is there any coordination mechanism to redistribute medicine, medical supplies and equipment to avoid waste at the county level?
2. On a scale from 0-10, how would you rate the supply chain in your county?
3. What has been done well regarding the supply chain in your county?
   1. How does it affect the performance of health systems?
4. What are the key challenges regarding the supply chain in your county? Please explore:
   1. Key indicators on measuring performance of supply chain (i.e. stockout issue of essential and non-essential medicines in health facilities in your county? If yes, please explore the magnitude and reasons for stockout, delay in delivery to health facilities, wastage and expiry of drugs, medical supplies and damage to equipment)
   2. How do these challenges affect the performance?
5. What is your suggestion to improve the supply chain in your county?
6. How feasible is your suggestion to make changes and why?

**III. KIIs for human resource manager**

1. **Procurement of human resources**

**C1. Questions related to hiring/retaining health personnel**

1. Please describe the hiring process of health personnel at public health facilities in your county. Please explore 5Ws and 1H (who, when, where, why, what, and how) when applicable.
   1. Do the hospitals and health centers have authorities to make decisions on hiring and laying off health staff
   2. Is the job description and eligibility publicly available?
   3. Is the selection process transparent?
   4. How does devolution affect the hiring and staff management process?
2. On a scale of 0 to 10, how would you rate the hiring process of health personnel in your county?
3. What has been done well in hiring health personnel in your county?
   1. How does it affect the performance of the health systems?
4. What are key challenges and gaps in hiring and retaining health personnel? Please explore:
   1. Over or under supply of health personnel
   2. Qualifications and skill-mix of personnel, whether they match the needs at health facilities?
   3. Factors that affect motivation and job satisfaction (i.e. salary determination and payment, promotion opportunities, training opportunities, and other monetary and non-monetary incentives)?
5. What is your suggestion to improve hiring and retaining health personnel?
6. How feasible is your suggestion to make changes and why?

**IV. KIIs for health providers**

1. **Supply and demand factors of health services:**

The following questions refer to key factors impacting the health system’s performance related to the supply of health services (including availability, access, and quality of health care) and the demand side (including affordability and acceptability of care).

**D1. Questions related to availability and accessibility**

1. Are health services provided at health facilities according to the county’s requirement? Please explore:
   1. What are the key services provided at various levels of health facilities, including lab tests?
   2. Are there major services missing in health facilities?
2. How accessible do you think health services are to the population in your county? Please explore the accessibility indicators:
   1. Percentage of the population covered within 5 km of the health facility
3. In a scale of 0-10, how would you rate the:
   1. availability of services
   2. accessibility of services?
4. What has been done well in your county regarding availability and accessibility?
   1. How does it affect the performance?
5. What are the key challenges regarding availability and accessibility of health services? Please explore:
   1. Physical access (remoteness)
   2. Availability of transport
   3. Funding disbursement issues
   4. Decision making process
6. What is your suggestion to improve the availability and accessibility, respectively?
7. How feasible is your suggestion to make changes and why?

**D2. Questions related to quality of care**

1. What are the current policies and interventions to improve the quality of care in your county?
2. On a scale of 0-10, how would you rate the quality of health care in your county?
3. What has been done well in your county to improve the quality of care?
   1. How does it affect the performance?
4. What are the key challenges affecting quality of care? Please explore:
   1. Qualification and technical skills of personnel and staff
   2. Existence of essential infrastructure
   3. Availability of drugs and equipment and prescription pattern (over prescription or under prescription, and drug quality)
   4. Responsiveness (respect) of personnel
5. What is your suggestion to improve the quality of care?
6. How feasible is your suggestion to make changes and why?

**D3. Questions related to affordability and acceptability of care**

1. Could you tell me how fees are charged at public and private health facilities for seeking care?
2. How would you assess the affordability and acceptability of care, respectively, in your county?
3. On a scale of 0-10, how would you rate the affordability and acceptability, respectively, in your county (high number means better affordability and acceptability)?
4. What has been done well to improve the affordability and acceptability of care? Please explore existence of (1) user fee exemption programs; (2) health insurance schemes; (3) voucher schemes; (4) cash transfer programs. For each program, please explore in more detail using 5Ws and 1H framework (Who, when, why, what, where, and how) where applicable, as well as population covered in each program.
   1. How do these programs affect the performance of health systems?
5. What are the major challenges in providing affordable and acceptable health services to the population in your county?
6. What is your suggestion to improve the affordability and acceptability of care?
7. How feasible is your suggestion to make changes and why?
8. If you have a chance to do three things, what will you do to improve the affordability and acceptability in your county?
9. **Use of inputs to provide health services**

This component asks questions regarding the health system design and how the latter affects the way in which inputs – drugs, equipment, personnel, information systems and financial resources – are used to provide health services. For example, health system design includes: community mobilization, referral systems, and drugs management.

1. Please describe how the health system is organized in your county including how the community is mobilized, number and types of health facilities, and collaboration with the private sector.
2. On a scale of 0 to 10, how would you rate the health service delivery in your county?
3. What has been done well in providing health services in your county?
   1. How does it affect the performance?
4. What are major challenges in providing health services?
   1. How do these challenges affect the performance?
5. What is your suggestion to improve the health service delivery?
6. How feasible is your suggestion to make changes and why?
7. If you have a chance to do three things, what will you do to improve health service delivery in your county?

**E1. Questions related to referral systems**

1. Is there a referral system? If yes, how does it work? Is it effective in directing patients to the most appropriate level of care?
2. On a scale of 0 to 10, how would you rate the referral system in your county?
3. What has been done well in providing health services in your county?
   1. How does it affect the performance?
4. What are major challenges of the referral system in your county? Please explore:
   1. How do these challenges affect the performance?
5. What is your suggestion to establish a more efficient and effective referral system?
6. How feasible is your suggestion to make changes and why?
7. If you have a chance to do three things, what will you do to improve the referral system in your county?

**E2. Questions related to community engagement**

1. How does the county engage the community in delivering health services, especially to improve maternal and child health, and/or communicable and non-communicable diseases?
2. How effective are these community programs in supporting health service delivery?
3. On a scale of 0 to 10, how would you rate the community engagement in your county?
4. What has been done well in engaging community in health service delivery in your county?
   1. How does it affect the performance?
5. What are major challenges in engaging the community for health service delivery?
   1. How do these challenges affect the performance?
6. What is your suggestion to improve the community engagement?
7. How feasible is your suggestion to make changes and why?

**E3. Question related to vertical programs**

1. Are there any vertical programs in the county? Examples (HIV/AIDs, TB, malaria, RMNCH) If there are, how is the funding from vertical programs disbursed (considering rigidities of funding and degree of service integration)?
2. How does the presence of vertical programs affect the service delivery in your county?
3. On a scale of 0 to 10, how would you rate the implementation of vertical programs and their impact on other health service delivery in your county?
4. What has been done well in implementing vertical programs and building synergy between vertical programs and other essential health services in your county?
   1. How does it affect the performance?
5. What are major challenges in implementing vertical programs and building synergy between vertical programs and other essential health services?
   1. How do these challenges affect the performance?
6. What is your suggestion to improve the community engagement?
7. How feasible is your suggestion to make changes and why?

**E4. Questions related to common issues on health input factors** Providing services requires service inputs, including human resources, drugs and medical products, medical equipment, and health information system.

1. On a scale of 0 to 10, how would you rate the degree to which your county uses these resources well to provide health services?
2. What has been done well on using these input resources in your county
   1. How does it affect the performance?
3. What are major challenges in using these input resources?
   1. How do these challenges affect the performance? Please explore:
      1. Human resources: inadequate staffing, absenteeism, inadequate supervision, skill-mix, staff turnover, motivations;
      2. Drugs: stock out, overstock and expired drugs, quality, counterfeit, and inappropriate use (generic vs brand);
      3. Equipment: inadequate or oversupply of equipment, capacity to operate equipment, maintenance of equipment;
      4. Information system: lack of information systems; relevance of information systems for decision making; and capacity to use information systems.
4. What is your suggestion to improve the use of these input factors?
5. How feasible is your suggestion to make changes and why?

**V. KII for Financial Department Manager**

**F1. Questions related to allocation of input factors (e.g. human resource, drugs, and equipment)**

1. How is the health budget allocated (i.e. by level of care: primary/secondary/tertiary level; by type of care: preventive vs curative care; by factors of expense: human resources, drugs, and equipment: by type costs: development versus recurrent costs)? What criteria are used to allocate budget for different services?
2. What is your opinion about the budget allocation this year?
   1. Do you think this is an effective way to allocate resources to maximize health outcomes?
3. On a scale of 0 to 10, how would you rate the resource allocation for health in your county?
4. What has been done well in allocating resources for health in your county?
   1. How does it affect the performance?
5. What are major challenges in allocating resources for health in your county?
   1. How do these challenges affect the performance?
6. What is your suggestion to improve the resource allocation for health in your county?
7. How feasible is your suggestion to make changes and why?

Stakeholder survey questionnaire

**Instruction:**

This survey is to assess the magnitude and causes of the inefficiencies in each component of the **county health systems**. The stakeholder survey is guided by the framework shown in Figure 4. The red boxes are the potential causes of (in)efficiencies of health system, which is the focus of this survey.


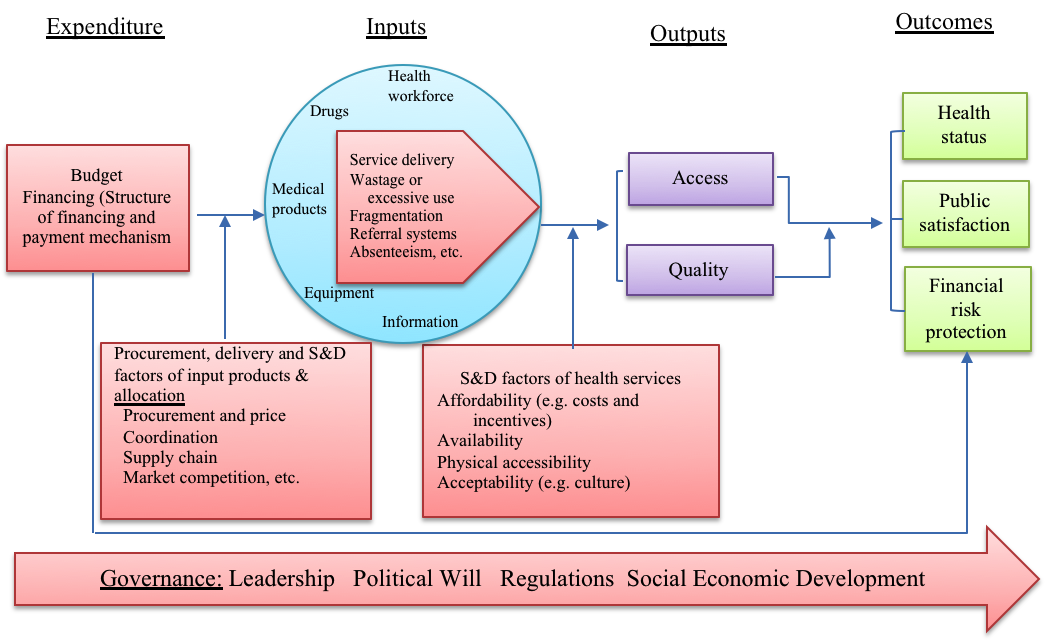


Figure 4. Potential inefficiencies in the health system

Note: S&D refers to supply and demand

We will ask a series of questions to understand your opinions regarding how much each factor impacts on the county health system’s performance in delivering health services and maximizing the population’s health. We will first ask your opinions regarding the existence of performance gaps in each of the five domains in the framework. We will then list potential sources of inefficiency, and for each of them, we will ask you how much each source impacts the county’s health system performance. The answer magnitude ranges from 0-5, where 0 means no efficiency gains, which suggests an optimal performance of the county health system, and 5 means the highest efficiency gains, suggesting that the county health system does not perform well and there is substantial scope for improvement.

**General information of participants**

County name: ________________________________________

Years in position (yrs): _______________________

**Inefficiencies in county health systems:** Please choose answers to each of the questions.

**1. What is your opinion about the statement that “there is inefficiency in governance and leadership for health”? ____**

1. Strongly disagree 2. Disagree 3. Neutral 4. Agree 5. Strongly agree

If the answer is 3, 4, or 5, please continue with 1.1.

- 1. **Now I am going to list different relevant factors that represent potential sources of inefficiency/non-performance. For each of them, please indicate the extent to which it affects the performance of the county health systems.**

| **Potential causes of inefficiencies/non-performance in governance and leadership for health** | **Extent to which this affects performance in your county** | | | | | |
| --- | --- | --- | --- | --- | --- | --- |
|  | [no efficiency gains (0) to substantial efficiency gains (5) if addressed], Please circle one answer | | | | | |
| Lack of political commitment at the county level | 0 | 1 | 2 | 3 | 4 | 5 |
| Poor leadership at the county level | 0 | 1 | 2 | 3 | 4 | 5 |
| Lack of or unclear local policies and strategies | 0 | 1 | 2 | 3 | 4 | 5 |
| Lack of detailed workplans | 0 | 1 | 2 | 3 | 4 | 5 |
| Lack of policy implementation capacity | 0 | 1 | 2 | 3 | 4 | 5 |
| Lack of implementation autonomy | 0 | 1 | 2 | 3 | 4 | 5 |
| Ambiguous roles and responsibilities | 0 | 1 | 2 | 3 | 4 | 5 |
| Unclear regulation rules | 0 | 1 | 2 | 3 | 4 | 5 |
| Inefficient communication and coordination | 0 | 1 | 2 | 3 | 4 | 5 |
| Existence of corruption | 0 | 1 | 2 | 3 | 4 | 5 |
| Insufficient supportive supervision | 0 | 1 | 2 | 3 | 4 | 5 |
| Lack of transparency in the decision-making process | 0 | 1 | 2 | 3 | 4 | 5 |
| Lack of accountability (monitoring and evaluation processes) | 0 | 1 | 2 | 3 | 4 | 5 |
| Others (describe)______________________________ | 0 | 1 | 2 | 3 | 4 | 5 |

**2. What is your opinion about the statement that “there is inefficiency in the budget development process”? _____**

1. Strongly disagree 2. Disagree 3. Neutral 4. Agree 5. Strongly agree

If the answer is 3, 4, or 5, please continue with 2.1

**2.1 Now I am going to list different relevant factors that represent potential sources of inefficiency/non-performance. For each of them, please indicate the extent to which it affects the performance of the county health systems.**

| **Potential causes of inefficiencies/non-performance the budget development process** | **Extent to which this affects performance in your county** | | | | | | |
| --- | --- | --- | --- | --- | --- | --- | --- |
|  | [no efficiency gains (0) to substantial efficiency gains (5) if addressed], Please circle one answer | | | | | | |
| Lengthy budget preparation cycle | 0 | 1 | 2 | 3 | 4 | 5 |  |
| Inappropriate budget preparation process | 0 | 1 | 2 | 3 | 4 | 5 |  |
| Lack of guidelines for budget preparation | 0 | 1 | 2 | 3 | 4 | 5 |  |
| Insufficient consultation with stakeholders | 0 | 1 | 2 | 3 | 4 | 5 |  |
| Lack of costing data for budgeting | 0 | 1 | 2 | 3 | 4 | 5 |  |
| Low capacity to develop a sector-wide budget | 0 | 1 | 2 | 3 | 4 | 5 |  |
| Lack of supervision and review of the final budget | 0 | 1 | 2 | 3 | 4 | 5 |  |
| Uncertainty related to external resources | 0 | 1 | 2 | 3 | 4 | 5 |  |
| Others (describe)______________________________ | 0 | 1 | 2 | 3 | 4 | 5 |  |

**3. What is your opinion about the statement that “there is inefficiency in budget execution”? ____**

1. Strongly disagree 2. Disagree 3. Neutral 4. Agree 5. Strongly agree

If the answer is 3, 4, or 5, please continue with 3.1

**3.1 Now I am going to list different relevant factors that represent potential sources of inefficiency/non-performance. For each of them, please indicate the extent to which it affects the performance of the county health systems.**

| **Potential causes of inefficiencies/non-performance in budget execution** | **Extent to which this affects performance in your county** | | | | | |
| --- | --- | --- | --- | --- | --- | --- |
|  | [no efficiency gains (0) to substantial efficiency gains (5) if addressed], Please circle one answer | | | | | |
| Rigid line-item budget | 0 | 1 | 2 | 3 | 4 | 5 |
| Lengthy process to disburse funding | 0 | 1 | 2 | 3 | 4 | 5 |
| Lengthy financial auditing process | 0 | 1 | 2 | 3 | 4 | 5 |
| Unavailability of funds when needed | 0 | 1 | 2 | 3 | 4 | 5 |
| Budget does not match needs | 0 | 1 | 2 | 3 | 4 | 5 |
| Poor capacity of designing and implementing projects/programs | 0 | 1 | 2 | 3 | 4 | 5 |
| Poor coordination within the health sector | 0 | 1 | 2 | 3 | 4 | 5 |
| Poor coordination with donors and/or the private sector | 0 | 1 | 2 | 3 | 4 | 5 |
| Insufficient autonomy of health facilities in using funding | 0 | 1 | 2 | 3 | 4 | 5 |
| Understaffing | 0 | 1 | 2 | 3 | 4 | 5 |
| Others ________________________________ | 0 | 1 | 2 | 3 | 4 | 5 |

**4. What is your opinion about the statement that “there is inefficiency in budget allocation”? _____**

1. Strongly disagree 2. Disagree 3. Neutral 4. Agree 5. Strongly agree

If the answer is 3, 4, or 5, please continue with 4.1.

**4.1 Now I am going to list different relevant factors that represent potential sources of inefficiency/non-performance. For each of them, please indicate the extent to which it affects the performance of the county health systems (multiple choice applies).**

| **Potential causes of inefficiencies/non-performance in budget allocation** | **Extent to which this affects performance in your county** | | | | | | |
| --- | --- | --- | --- | --- | --- | --- | --- |
|  | [no efficiency gains (0) to substantial efficiency gains (5) if addressed], Please circle one answer | | | | | | |
| Complicated political consideration | 0 | 1 | 2 | 3 | 4 | 5 |  |
| Inappropriate budget process | 0 | 1 | 2 | 3 | 4 | 5 |  |
| Limited information for priority setting | 0 | 1 | 2 | 3 | 4 | 5 |  |
| Poor coordination within the health sector | 0 | 1 | 2 | 3 | 4 | 5 |  |
| Poor coordination with donors and/or the private sector | 0 | 1 | 2 | 3 | 4 | 5 |  |
| Others (describe)___________________________ | 0 | 1 | 2 | 3 | 4 | 5 |  |

**5. What is your opinion about the statement that “there is inefficiency in paying health facilities”?** ____

1. Strongly disagree 2. Disagree 3. Neutral 4. Agree 5. Strongly agree

If the answer is 3, 4, or 5, please continue with 5.1

**5.1 Now I am going to list different relevant factors that represent potential sources of inefficiency/non-performance. For each of them, please indicate the extent to which it affects the performance of the county health systems.**

| **Potential causes of inefficiencies/non-performance in paying health facilities** | **Extent to which this affects performance in your county** | | | | | |
| --- | --- | --- | --- | --- | --- | --- |
|  | [no efficiency gains (0) to substantial efficiency gains (5) if addressed], Please circle one answer | | | | | |
| Rigid payment mechanisms (i.e. line-item budget) | 0 | 1 | 2 | 3 | 4 | 5 |
| Delays in payments | 0 | 1 | 2 | 3 | 4 | 5 |
| Insufficient financial incentives for providing more services | 0 | 1 | 2 | 3 | 4 | 5 |
| Insufficient financial incentives for providing better services | 0 | 1 | 2 | 3 | 4 | 5 |
| Inadequate monitoring and evaluation of payment mechanisms | 0 | 1 | 2 | 3 | 4 | 5 |
| Others ________________________________ | 0 | 1 | 2 | 3 | 4 | 5 |

**6. What is your opinion about the statement that “there is inefficiency in procuring medicine, supplies and equipment”?** _____

1. Strongly disagree 2. Disagree 3. Neutral 4. Agree 5. Strongly agree

If the answer is 3, 4, or 5, please continue 6.1

**6.1 Now I am going to list different relevant factors that represent potential sources of inefficiency/non-performance. For each of them, please indicate the extent to which it affects the performance of the county health systems.**

| **Potential causes of inefficiencies/non-performance in procuring medicine, supplies and equipment** | **Extent to which this affects performance in your county** | | | | | |
| --- | --- | --- | --- | --- | --- | --- |
|  | [no efficiency gains (0) to substantial efficiency gains if addressed (5)] | | | | | |
| Lack of or unclear procurement policy | 0 | 1 | 2 | 3 | 4 | 5 |
| Rigid procurement policy | 0 | 1 | 2 | 3 | 4 | 5 |
| Lack of or poor capacity of the procurement committee | 0 | 1 | 2 | 3 | 4 | 5 |
| Lack of skills to plan and budget inputs needed | 0 | 1 | 2 | 3 | 4 | 5 |
| Poor mechanism to determine types and amount of product for procurement | 0 | 1 | 2 | 3 | 4 | 5 |
| Limited competition of product suppliers | 0 | 1 | 2 | 3 | 4 | 5 |
| Low and limited negotiation power and capacity | 0 | 1 | 2 | 3 | 4 | 5 |
| Insufficient price control and setting capacity | 0 | 1 | 2 | 3 | 4 | 5 |
| Lack of procurement autonomy at health facilities | 0 | 1 | 2 | 3 | 4 | 5 |
| Resources are used for other purposes | 0 | 1 | 2 | 3 | 4 | 5 |
| Limited domestic production | 0 | 1 | 2 | 3 | 4 | 5 |
| Others (specify)___________________ | 0 | 1 | 2 | 3 | 4 | 5 |

**7. What is your opinion about the statement that “there is inefficiency in hiring and paying health personnel”? ____**

1. Strongly disagree 2. Disagree 3. Neutral 4. Agree 5. Strongly agree

If the answer is 3, 4, or 5, please continue with 7.1

**7.1 Now I am going to list different relevant factors that represent potential sources of inefficiency/non-performance. For each of them, please indicate the extent to which it affects the performance of the county health systems.**

| **Potential causes of inefficiencies/non-performance in hiring and paying health personnel** | **Extent to which this affects performance in your county** | | | | | | |
| --- | --- | --- | --- | --- | --- | --- | --- |
|  | [no efficiency gains (0) to substantial efficiency gains if addressed (5)] | | | | | | |
| Lack of or unclear staff hiring policy | 0 | 1 | 2 | 3 | 4 | 5 |  |
| Lack of autonomy in hiring/laying off staff at health facilities | 0 | 1 | 2 | 3 | 4 | 5 |  |
| Lack of transparency in the hiring process | 0 | 1 | 2 | 3 | 4 | 5 |  |
| Unfair salary structure (e.g. based on age rather than merit) | 0 | 1 | 2 | 3 | 4 | 5 |  |
| Issues with funds flow | 0 | 1 | 2 | 3 | 4 | 5 |  |
| Lack of professional career development opportunities | 0 | 1 | 2 | 3 | 4 | 5 |  |
| Insufficient training and educational opportunities | 0 | 1 | 2 | 3 | 4 | 5 |  |
| Limited supply of medical graduates | 0 | 1 | 2 | 3 | 4 | 5 |  |
| Others (specify)___________________ | 0 | 1 | 2 | 3 | 4 | 5 |  |

**8. What is your opinion about the statement that “there is inefficiency in making health services available for and accessible to people”? ____**

1. Strongly disagree 2. Disagree 3. Neutral 4. Agree 5. Strongly agree

If the answer is 3, 4, or 5, please continue with 8.1

**8.1 Now I am going to list different relevant factors that represent potential sources of inefficiency/non-performance. For each of them, please indicate the extent to which it affects the performance of the county health systems.**

| **Potential causes of inefficiencies/non-performance in making health services available for and accessible to people** | **Extent to which this affects performance in your county** | | | | | |
| --- | --- | --- | --- | --- | --- | --- |
|  | [no efficiency gains (0) to substantial efficiency gains if addressed (5)] | | | | | |
| Insufficient health facilities | 0 | 1 | 2 | 3 | 4 | 5 |
| Poor location of health facilities | 0 | 1 | 2 | 3 | 4 | 5 |
| Insufficient resources to provide needed services | 0 | 1 | 2 | 3 | 4 | 5 |
| Poor management of resources at health facilities | 0 | 1 | 2 | 3 | 4 | 5 |
| Physical challenges due to the remoteness | 0 | 1 | 2 | 3 | 4 | 5 |
| Poor supply chain of drugs | 0 | 1 | 2 | 3 | 4 | 5 |
| Lack of essential lab tests and image services | 0 | 1 | 2 | 3 | 4 | 5 |
| Limited staff skills (i.e. poor diagnosis, and treatment) | 0 | 1 | 2 | 3 | 4 | 5 |
| Poor community mobilization | 0 | 1 | 2 | 3 | 4 | 5 |
| Insufficient outreach programs | 0 | 1 | 2 | 3 | 4 | 5 |
| Health provider overburdened with data reporting | 0 | 1 | 2 | 3 | 4 | 5 |
| Others (specify)___________________ | 0 | 1 | 2 | 3 | 4 | 5 |

**9. What is your opinion about the statement that “there is inefficiency in providing affordable and acceptable services to people”?**

1. Strongly disagree 2. Disagree 3. Neutral 4. Agree 5. Strongly agree

If the answer is 3, 4, or 5, please continue with 9.1

**9.1 Now I am going to list different relevant factors that represent potential sources of inefficiency/non-performance. For each of them, please indicate the extent to which it affects the performance of the county health systems.**

| **Potential causes of inefficiencies/non-performance in providing affordable and acceptable services to people** | **Extent to which this affects performance in your county** | | | | | |  |
| --- | --- | --- | --- | --- | --- | --- | --- |
|  | [no efficiency gains (0) to substantial efficiency gains if addressed (5)] | | | | | |  |
| User fees to patients | 0 | 1 | 2 | 3 | 4 | 5 | |
| Under the table payment | 0 | 1 | 2 | 3 | 4 | 5 | |
| Poor economic status of the community | 0 | 1 | 2 | 3 | 4 | 5 | |
| Lack of financial protection mechanisms | 0 | 1 | 2 | 3 | 4 | 5 | |
| High illiteracy in the community | 0 | 1 | 2 | 3 | 4 | 5 | |
| Insufficient health education | 0 | 1 | 2 | 3 | 4 | 5 | |
| Cultural barriers | 0 | 1 | 2 | 3 | 4 | 5 | |
| Others (specify)___________________ | 0 | 1 | 2 | 3 | 4 | 5 | |

**10. What is your opinion about the statement that “there is an inefficiency in how health delivery system is organized”?**

1. Strongly disagree 2. Disagree 3. Neutral 4. Agree 5. Strongly agree

If the answer is 3, 4, or 5, please continue with 10.1

**10.1 Now I am going to list different relevant factors that represent potential sources of inefficiency/non-performance. For each of them, please indicate the extent to which it affects the performance of the county health systems.**

| **Potential causes of inefficiencies/non-performance in how health delivery system is organized** | **Extent to which this affects performance in your county** | | | | | |
| --- | --- | --- | --- | --- | --- | --- |
|  | [no efficiency gains (0) to substantial efficiency gains if addressed (5)] | | | | | |
| Imbalance in the number of primary care and higher-level care facilities | 0 | 1 | 2 | 3 | 4 | 5 |
| Poor referral systems | 0 | 1 | 2 | 3 | 4 | 5 |
| Fragmented health providers | 0 | 1 | 2 | 3 | 4 | 5 |
| Poor community engagement | 0 | 1 | 2 | 3 | 4 | 5 |
| Poor integration of vertical programs in the health systems | 0 | 1 | 2 | 3 | 4 | 5 |
| Overuse of brand drugs | 0 | 1 | 2 | 3 | 4 | 5 |
| Overuse of medical supplies | 0 | 1 | 2 | 3 | 4 | 5 |
| Overuse of lab test and equipment | 0 | 1 | 2 | 3 | 4 | 5 |
| Wastage of drugs (expired drugs) | 0 | 1 | 2 | 3 | 4 | 5 |
| Use of low quality or counterfeit drugs | 0 | 1 | 2 | 3 | 4 | 5 |
| Inappropriate use of drugs | 0 | 1 | 2 | 3 | 4 | 5 |
| Poor maintenance of equipment | 0 | 1 | 2 | 3 | 4 | 5 |
| Low capacity to operation equipment | 0 | 1 | 2 | 3 | 4 | 5 |
| Absenteeism of health care workers | 0 | 1 | 2 | 3 | 4 | 5 |
| Inadequate motivation and incentives for providers | 0 | 1 | 2 | 3 | 4 | 5 |
| Insufficient data for decision making | 0 | 1 | 2 | 3 | 4 | 5 |
| Low capacity to use existing data for decision making | 0 | 1 | 2 | 3 | 4 | 5 |
| Lack of quality accreditation and supervision | 0 | 1 | 2 | 3 | 4 | 5 |
| Others (specify)___________________ | 0 | 1 | 2 | 3 | 4 | 5 |

**Questions on public finance management (PFM)**

**Budget formulation**

**1. Do you receive a budget?**

1. Yes 2. No

If the answer is “yes”, please continue with 1.1

**1.1 Do you know how much your facility has been allocated in the budget?**

1. Yes 2. No

If the answer is “yes”, please continue with 1.2

**1.2. Does your final budget reflect your original plan?**

1. Strongly disagree 2. Disagree 3. Neutral 4. Agree 5. Strongly agree

If the answer is 1-3, please continue with 1.3

**1.3. Please identify a reason why your final budget does not reflect the original plan**

| **Why does your budget not reflect your original plan?** | **Select which applies*** | | | | |
| --- | --- | --- | --- | --- | --- |
| Original plans are not realistic | 1 | 2 | 3 | 4 | 5 |
| Budget allocations are changed by CHMT without your consultation | 1 | 2 | 3 | 4 | 5 |
| Budget allocations are reprioritized by county assembly. Changes are made without your consultation | 1 | 2 | 3 | 4 | 5 |
| Others (describe)_________________________ | 1 | 2 | 3 | 4 | 5 |

*1. Strongly disagree 2. Disagree 3. Neutral 4. Agree 5. Strongly agree

**1.4. It is cumbersome to deal with multiple funding sources during the planning and budgeting process.**

1. Strongly disagree 2. Disagree 3. Neutral 4. Agree 5. Strongly agree

**1.5. You don’t have a comprehensive plan because of multiple fragmented funding sources that are off-budget.**

1. Strongly disagree 2. Disagree 3. Neutral 4. Agree 5. Strongly agree

**Budget execution**

**2.1 We know when and what goods and services we will receive to provide services at our facility**

1. Strongly disagree 2. Disagree 3. Neutral 4. Agree 5. Strongly agree

**2.2 The funds released reflect our approved budget**

1. Strongly disagree 2. Disagree 3. Neutral 4. Agree 5. Strongly agree

**2.3. We are informed when funds are available for the implementation of activities**

1. Strongly disagree 2. Disagree 3. Neutral 4. Agree 5. Strongly agree

**2.4 Funds from fees and charges are sent back to the county revenue fund**

1. Yes 2. No

**2.5 We have sufficient autonomy to execute the budget well**

1. Strongly disagree 2. Disagree 3. Neutral 4. Agree 5. Strongly agree

**2.6. Budget releases are usually on time**

1. Strongly disagree 2. Disagree 3. Neutral 4. Agree 5. Strongly agree

**2.7. What funding sources are easiest to deploy**

| **What funding sources are easiest to deploy** | **Select which applies*** | | | | | |
| --- | --- | --- | --- | --- | --- | --- |
| Funding from the equitable share | 1 | 2 | 3 | 4 | 5 |  |
| Conditional grant funding | 1 | 2 | 3 | 4 | 5 |  |
| Funding from own source revenues (in case they are retained) | 1 | 2 | 3 | 4 | 5 |  |
| Funding from Development Partners (DPs) | 1 | 2 | 3 | 4 | 5 |  |
| Which DPs?______________________________ | 1 | 2 | 3 | 4 | 5 |  |

*1. Strongly disagree 2. Disagree 3. Neutral 4. Agree 5. Strongly agree

**Budget evaluation**

**3.1 Your performance has affected your budget allocation in the next year**

1. Strongly disagree 2. Disagree 3. Neutral 4. Agree 5. Strongly agree

**3.2 The budget evaluation process takes into consideration changing needs as it informs subsequent budgets**

1. Strongly disagree 2. Disagree 3. Neutral 4. Agree 5. Strongly agree

**3.3 There are sanctions in place to penalize poor service quality during the budget evaluation**

1. Strongly disagree 2. Disagree 3. Neutral 4. Agree 5. Strongly agree

**3.4 Auditors are unable to hold counties accountable because they don’t track to which facilities goods and services were delivered (they only capture the financial transaction at the county level)**

1. Yes 2. No

# Appendix 2. Appendix Tables

Table A 1. Potential indicators for improvement in governance

| **Indicators** | **County 1** | **County 2** | **County 3** | **County 4** | **County 5** |
| --- | --- | --- | --- | --- | --- |
| Lack of political commitment at the county level | 2.70 | 2.25 | 3.56 | 2.25 | 3.63 |
| Poor leadership at the county level | 2.10 | 2.75 | 3.30 | 2.33 | 3.32 |
| Lack of or unclear local policies and strategies | 1.70 | 2.75 | 3.38 | 2.60 | 3.37 |
| Lack of detailed workplans | 1.30 | 3.25 | 2.67 | 2.25 | 2.72 |
| Lack of policy implementation capacity | 2.00 | 3.75 | 3.11 | 2.90 | 2.89 |
| Lack of implementation autonomy | 3.10 | 4.00 | 3.58 | 3.33 | 3.26 |
| Ambiguous roles and responsibilities | 2.20 | 2.50 | 3.44 | 2.57 | 2.89 |
| Unclear regulation rules | 1.50 | 2.00 | 2.81 | 2.19 | 2.74 |
| Inefficient communication and coordination | 2.55 | 2.50 | 3.11 | 2.75 | 2.65 |
| Existence of corruption | 3.25 | 4.25 | 3.41 | 2.52 | 3.67 |
| Insufficient supportive supervision | 2.50 | 2.25 | 3.44 | 2.48 | 1.72 |
| Lack of transparency in the decision-making process | 2.60 | 2.00 | 3.44 | 2.10 | 2.58 |
| Lack of accountability (monitoring and evaluation processes) | 2.60 | 2.50 | 3.33 | 2.30 | 3.00 |
| Mean | 2.32 | 2.83 | 3.28 | 2.51 | 2.96 |

Note: Green indicates limited scope for improving inefficiency, and orange indicates the relatively high potential of savings from improved efficiency.

Table A 2.Potential indicators for improvement in budget preparation

| **Indicators** | County 1 | County 2 | County 3 | County 4 | County 5 |
| --- | --- | --- | --- | --- | --- |
| Lengthy budget preparation cycle | 2.67 | 1.75 | 2.96 | 3.11 | 3.07 |
| Inappropriate budget preparation process | 2.10 | 2.75 | 3.12 | 2.33 | 2.87 |
| Lack of guidelines for budget preparation | 1.73 | 3.00 | 2.42 | 2.22 | 2.27 |
| Insufficient consultation with stakeholders | 2.50 | 3.50 | 3.58 | 2.94 | 3.07 |
| Lack of costing data for budgeting | 3.08 | 3.75 | 3.00 | 2.83 | 3.40 |
| Low capacity to develop a sector-wide budget | 2.55 | 3.25 | 3.36 | 2.41 | 3.19 |
| Lack of supervision and review of the final budget | 3.00 | 4.00 | 3.23 | 2.56 | 2.81 |
| Uncertainty related to external resources | 2.30 | 4.00 | 3.32 | 3.50 | 2.00 |
| Average | 2.49 | 3.25 | 3.12 | 2.74 | 2.84 |

Note: Green indicates limited scope for improving inefficiency, and orange indicates the relatively high potential of savings from improved efficiency.

Table A 3. Potential indicators for improvement in budget execution

| **Indicators** | County 1 | County 2 | County 3 | County 4 | County 5 |
| --- | --- | --- | --- | --- | --- |
| Rigid line-item budget | 2.58 | 2.00 | 2.68 | 1.94 | 3.48 |
| Lengthy process to disburse funding | 3.42 | 3.33 | 4.15 | 2.89 | 3.88 |
| Lengthy financial auditing process | 2.42 | 2.17 | 3.00 | 2.00 | 3.33 |
| Unavailability of funds when needed | 3.23 | 3.00 | 4.54 | 3.06 | 4.25 |
| Budget does not match needs | 3.38 | 2.17 | 4.23 | 3.89 | 3.22 |
| Poor capacity of designing and implementing projects/programs | 2.83 | 2.67 | 3.62 | 3.45 | 3.00 |
| Poor coordination within the health sector | 2.42 | 2.00 | 3.04 | 2.84 | 2.92 |
| Poor coordination with donors and/or the private sector | 2.08 | 1.60 | 2.20 | 3.00 | 2.96 |
| Insufficient autonomy of health facilities in using funding | 2.58 | 1.75 | 3.96 | 3.63 | 3.21 |
| Understaffing | 3.46 | 4.00 | 4.19 | 3.22 | 4.38 |
| Average | 2.84 | 2.47 | 3.56 | 2.99 | 3.46 |

Note: Green indicates limited scope for improving inefficiency, and orange indicates the relatively high potential of savings from improved efficiency.

Table A 4. Potential indicators for improvement in budget allocation

| **Indicators** | County 1 | County 2 | County 3 | County 4 | County 5 |
| --- | --- | --- | --- | --- | --- |
| Complicated political consideration | 3.08 | 3.20 | 3.71 | 3.59 | 3.27 |
| Inappropriate budget process | 3.23 | 2.60 | 2.86 | 3.50 | 2.83 |
| Limited information for priority setting | 2.92 | 2.20 | 3.41 | 3.06 | 3.00 |
| Poor coordination within the health sector | 2.92 | 2.00 | 2.50 | 3.53 | 3.22 |
| Poor coordination with donors and/or the private sector | 2.42 | 2.20 | 2.39 | 3.13 | 3.39 |
| Average | 2.91 | 2.44 | 2.97 | 3.36 | 3.14 |

Note: Green indicates limited scope for improving inefficiency, and orange indicates the relatively high potential of savings from improved efficiency.

Table A 5.Potential indicators for improvement on paying health facilities in five counties

| **Indicators** | **County 1** | **County 2** | **County 3** | **County 4** | **County 5** |
| --- | --- | --- | --- | --- | --- |
| Rigid payment mechanisms (i.e. line-item budget) | 3.50 | 3.20 | 3.72 | 2.48 | 3.50 |
| Delays in payments | 3.67 | 3.80 | 4.38 | 2.86 | 3.95 |
| Insufficient financial incentives for providing more services | 4.29 | 3.00 | 3.96 | 3.19 | 3.95 |
| Insufficient financial incentives for providing better services | 4.25 | 2.60 | 4.08 | 3.19 | 3.77 |
| Inadequate monitoring and evaluation of payment mechanisms | 2.75 | 3.60 | 3.48 | 2.86 | 3.62 |
| Average | 3.69 | 3.24 | 3.92 | 2.92 | 3.76 |

Note: Green indicates limited scope for improving inefficiency, and orange indicates the relatively high potential of savings from improved efficiency.

Table A 6. Potential indicators for improvement in procuring medicine, supplies and equipment in five counties

| **Indicators** | **County 1** | **County 2** | **County 3** | **County 4** | **County 5** |
| --- | --- | --- | --- | --- | --- |
| Lack of or unclear procurement policy | 3.00 | 2.20 | 2.56 | 2.50 | 2.68 |
| Rigid procurement policy | 3.38 | 2.40 | 3.16 | 2.58 | 3.32 |
| Lack of or poor capacity of the procurement committee | 3.43 | 3.20 | 2.48 | 2.26 | 3.16 |
| Lack of skills to plan and budget inputs needed | 1.86 | 1.60 | 2.48 | 2.26 | 2.39 |
| Poor mechanism to determine types and amount of product for procurement | 2.29 | 2.60 | 2.56 | 2.37 | 2.74 |
| Limited competition of product suppliers | 2.86 | 1.80 | 2.50 | 2.21 | 2.42 |
| Low and limited negotiation power and capacity | 3.00 | 1.20 | 2.50 | 2.32 | 2.89 |
| Insufficient price control and price setting capacity | 2.83 | 2.80 | 2.88 | 1.84 | 3.16 |
| Lack of procurement autonomy at health facilities | 4.00 | 2.60 | 4.08 | 3.11 | 3.58 |
| Resources are used for other purposes | 2.43 | 1.00 | 3.35 | 4.12 | 2.90 |
| Limited domestic production | 3.14 | 3.60 | 3.45 | 2.47 | 3.10 |
| Average | 2.93 | 2.27 | 2.91 | 2.55 | 2.94 |

Note: Green indicates limited scope for improving inefficiency, and orange indicates the relatively high potential of savings from improved efficiency.

Table A 7. Potential indicators for improvement on human resources in five counties

| **Indicators** | **County 1** | **County 2** | **County 3** | **County 4** | **County 5** |
| --- | --- | --- | --- | --- | --- |
| Lack of or unclear staff hiring policy | 3.30 | 3.17 | 3.28 | 3.13 | 3.05 |
| Lack of autonomy in hiring/laying off staff at health facilities | 3.20 | 1.67 | 3.42 | 3.43 | 3.90 |
| Lack of transparency in the hiring process | 3.80 | 1.33 | 3.72 | 3.13 | 3.00 |
| Unfair salary structure (e.g. based on age rather than merit) | 2.40 | 2.67 | 2.69 | 2.93 | 2.55 |
| Issues with funds flow | 2.55 | 2.50 | 3.31 | 3.53 | 3.48 |
| Lack of professional career development opportunities | 3.10 | 2.33 | 3.58 | 3.29 | 3.05 |
| Insufficient training and educational opportunities | 3.11 | 2.83 | 3.80 | 3.50 | 3.09 |
| Limited supply of medical graduates | 3.00 | 2.17 | 2.77 | 2.40 | 2.00 |
| Average | 3.06 | 2.33 | 3.32 | 3.17 | 3.02 |

Note: Green indicates limited scope for improving inefficiency, and orange indicates the relatively high potential of savings from improved efficiency.

Table A 8. Potential indicators for improvement on availability and accessibility of health care

| **Indicators** | **County 1** | **County 2** | **County 3** | **County 4** | **County 5** |
| --- | --- | --- | --- | --- | --- |
| Insufficient health facilities | 2.44 | 3.25 | 2.12 | 3.11 | 2.24 |
| Poor location of health facilities | 2.00 | 3.50 | 2.24 | 2.18 | 2.05 |
| Insufficient resources to provide needed services | 3.44 | 4.25 | 4.00 | 3.47 | 3.85 |
| Poor management of resources at health facilities | 2.56 | 2.20 | 2.20 | 3.06 | 2.62 |
| Physical challenges due to the remoteness | 3.50 | 4.00 | 2.00 | 3.44 | 1.81 |
| Poor supply chain of drugs | 2.88 | 3.20 | 2.64 | 3.33 | 3.25 |
| Lack of essential lab tests and imaging services | 3.75 | 3.20 | 4.24 | 3.06 | 3.40 |
| Limited staff skills (i.e. poor diagnosis, and treatment) | 2.88 | 3.60 | 2.52 | 2.75 | 2.00 |
| Poor community mobilization | 3.13 | 2.25 | 2.52 | 2.28 | 2.33 |
| Insufficient outreach programs | 2.89 | 2.80 | 3.12 | 2.61 | 2.71 |
| Health provider overburdened with data reporting | 3.11 | 3.20 | 4.04 | 2.47 | 4.29 |
| Average | 2.96 | 3.22 | 2.88 | 2.89 | 2.78 |

Note: Green indicates limited scope for improving inefficiency, and orange indicates the relatively high potential of savings from improved efficiency.

Table A 9. Inefficiency in providing affordable and culturally acceptable health care

| **Indicators** | **County 1** | **County 2** | **County 3** | **County 4** | **County 5** |
| --- | --- | --- | --- | --- | --- |
| User fees to patients | 2.14 | 3.67 | 2.50 | 2.15 | 2.88 |
| Under the table payment | 1.29 | 1.75 | 2.32 | 1.69 | 2.15 |
| Poor economic status of the community | 4.25 | 5.00 | 2.86 | 2.56 | 3.23 |
| Lack of financial protection mechanisms | 3.43 | 3.80 | 3.14 | 3.44 | 2.95 |
| High illiteracy in the community | 3.50 | 4.20 | 2.50 | 3.00 | 2.64 |
| Insufficient health education | 3.63 | 4.40 | 3.14 | 3.31 | 2.86 |
| Cultural barriers | 4.00 | 4.60 | 1.95 | 3.27 | 2.48 |
| Average | 3.18 | 3.92 | 2.63 | 2.77 | 2.74 |

Note: Green indicates limited scope for improving inefficiency, and orange indicates the relatively high potential of savings from improved efficiency.

Table A 10. Inefficiency on how the health delivery system is organized

| **Indicators** | **County 1** | **County 2** | **County 3** | **County 4** | **County 5** |
| --- | --- | --- | --- | --- | --- |
| Imbalance in the number of primary care and higher-level care facilities | 2.33 | 3.40 | 2.78 | 2.87 | 2.79 |
| Poor referral systems | 2.33 | 4.00 | 2.96 | 2.69 | 3.14 |
| Fragmented health providers | 2.63 | 2.20 | 2.82 | 2.75 | 3.00 |
| Poor community engagement | 2.11 | 3.20 | 2.78 | 2.00 | 3.05 |
| Poor integration of vertical programs in the health systems | 3.00 | 2.80 | 3.13 | 2.75 | 2.62 |
| Overuse of brand drugs | 1.44 | 3.00 | 2.30 | 2.00 | 2.05 |
| Overuse of medical supplies | 2.22 | 3.00 | 2.13 | 1.94 | 1.60 |
| Overuse of lab test and equipment | 1.89 | 3.40 | 2.17 | 2.24 | 1.84 |
| Wastage of drugs (expired drugs) | 2.11 | 2.60 | 2.65 | 2.24 | 1.95 |
| Use of low quality or counterfeit drugs | 1.89 | 2.50 | 2.30 | 2.12 | 1.65 |
| Inappropriate use of drugs | 1.44 | 3.20 | 1.62 | 2.47 | 1.15 |
| Poor maintenance of equipment | 3.00 | 3.20 | 3.43 | 3.53 | 3.45 |
| Low capacity to operation equipment | 2.78 | 2.00 | 3.00 | 3.25 | 2.86 |
| Absenteeism of health care workers | 1.00 | 2.80 | 2.13 | 2.13 | 1.80 |
| Insufficient data for decision making | 2.11 | 1.60 | 2.29 | 3.27 | 2.45 |
| Low capacity to use existing data for decision making | 2.56 | 2.80 | 2.82 | 3.00 | 2.57 |
| Lack of quality accreditation and supervision | 3.00 | 2.40 | 2.91 | 2.33 | 2.95 |
| Average | 2.23 | 2.83 | 2.60 | 2.56 | 2.41 |

Note: Green indicates limited scope for improving inefficiency, and orange indicates the relatively high potential of savings from improved efficiency.
